# Equity considerations in clinical practice guidelines for traumatic brain injury and the criminal justice system: A systematic review

**DOI:** 10.1371/journal.pmed.1004418

**Published:** 2024-08-12

**Authors:** Zoe Colclough, Maria Jennifer Estrella, Julie Michele Joyce, Sara Hanafy, Jessica Babineau, Angela Colantonio, Vincy Chan

**Affiliations:** 1 Department of Forensic Science, University of Toronto, Mississauga, Canada; 2 Department of Occupational Science and Occupational Therapy, University of Toronto, Toronto, Canada; 3 Rehabilitation Sciences Institute, University of Toronto, Toronto, Canada; 4 KITE Research Institute-Toronto Rehabilitation Institute, University Health Network, Toronto, Canada; 5 Library and Information Services, University Health Network, Toronto, Canada; 6 The Institute for Education Research, University Health Network, Toronto, Canada; 7 Institute of Health Policy, Management and Evaluation, University of Toronto, Toronto, Canada; 8 Dalla Lana School of Public Health, University of Toronto, Toronto, Canada

## Abstract

**Background:**

Traumatic brain injury (TBI) is disproportionately prevalent among individuals who intersect or are involved with the criminal justice system (CJS). In the absence of appropriate care, TBI-related impairments, intersecting social determinants of health, and the lack of TBI awareness in CJS settings can lead to lengthened sentences, serious disciplinary charges, and recidivism. However, evidence suggests that most clinical practice guidelines (CPGs) overlook equity and consequently, the needs of disadvantaged groups. As such, this review addressed the research question “To what extent are (1) intersections with the CJS considered in CPGs for TBI, (2) TBI considered in CPGs for CJS, and (3) equity considered in CPGs for CJS?”.

**Methods and findings:**

CPGs were identified from electronic databases (MEDLINE, Embase, CINAHL, PsycINFO), targeted websites, Google Search, and reference lists of identified CPGs on November 2021 and March 2023 (CPGs for TBI) and May 2022 and March 2023 (CPGs for CJS). Only CPGs for TBI or CPGs for CJS were included. We calculated the proportion of CPGs that included TBI- or CJS-specific content, conducted a qualitative content analysis to understand how evidence regarding TBI and the CJS was integrated in the CPGs, and utilised equity assessment tools to understand if and how equity was considered. Fifty-seven CPGs for TBI and 6 CPGs for CJS were included in this review. Fourteen CPGs for TBI included information relevant to the CJS, but only 1 made a concrete recommendation to consider legal implications during vocational evaluation in the forensic context. Two CPGs for CJS acknowledged the prevalence of TBI among individuals in prison and one specifically recommended considering TBI during health assessments. Both CPGs for TBI and CPGs for CJS provided evidence specific to a single facet of the CJS, predominantly in policing and corrections. The use of equity best practices and the involvement of disadvantaged groups in the development process were lacking among CPGs for CJS. We acknowledge limitations of the review, including that our searches were conducted in English language and thus, we may have missed other non-English language CPGs in this review. We further recognise that we are unable to comment on evidence that is not integrated in the CPGs, as we did not systematically search for research on individuals with TBI who intersect with the CJS, outside of CPGs.

**Conclusions:**

Findings from this review provide the foundation to consider CJS involvement in CPGs for TBI and to advance equity in CPGs for CJS. Conducting research, including investigating the process of screening for TBI with individuals who intersect with all facets of the CJS, and utilizing equity assessment tools in guideline development are critical steps to enhance equity in healthcare for this disadvantaged group.

## Abstract 2

Zoe Colclough and co-authors examine how equity is considered in the development of clinical practice guidelines for traumatic brain injury for individuals within the criminal justice system.

## Author summary

**Why was this study done?:**

- Traumatic brain injury (TBI) is more common among individuals involved with the criminal justice system (CJS) than the general population, often resulting in longer prison sentences, serious disciplinary charges, and repeated future conflicts with the CJS.
- Although individuals with TBI who are involved with the CJS must receive care that meets their needs, studies show that most clinical practice guidelines (CPGs)—designed to guide care—tend to overlook equity and disadvantaged groups, instead focussing on the effectiveness or cost-effectiveness of care.
- As a first step to improving equitable care, this study assessed existing CPGs for TBI and CPGs for CJS to see if and how (1) evidence regarding individuals who intersect with the CJS is included in CPGs for TBI and evidence regarding TBI is integrated in CPGs for CJS; and (2) equity is considered in CPGs for CJS.

**What did the researchers do and find?:**

- We used available electronic databases (MEDLINE, Embase, CINAHL, PsycINFO), targeted websites, Google search, and reference lists for CPGs for TBI and CPGs for CJS.
- We documented their characteristics, reviewed their content, and assessed whether equity was considered using checklists focused on equity and disadvantaged groups.
- We found that 14 out of 57 CPGs for TBI referenced individuals who are involved with the CJS and 2 out of 6 CPGs for CJS referenced TBI.
- Practices to ensure that equity is considered, such as the involvement of disadvantaged groups when developing CPGs were lacking in CPGs for CJS.

**What do these findings mean?:**

- Findings from this review suggest that consideration of equity is lacking in the development of CPGs for TBI for those within the CJS and provide the foundation to consider CJS involvement in CPGs for TBI and to advance equity in CPGs for CJS.
- There is a critical need for further research into screening processes for TBI with individuals who intersect with all facets of the CJS, and into the benefits of equity assessment tools in guideline development to enhance equity in healthcare for this disadvantaged group
- Unfortunately, we cannot comment on how much of the existing evidence regarding TBI or individuals who are involved with the CJS remains unintegrated in existing CPGs, as our search did not include research papers on TBI and the CJS, outside of CPGs.

## Introduction

Traumatic brain injury (TBI) affects 69 million people every year [1] and may cause long-term challenges in behaviour, cognition, and communication [2] with functional limitations in various areas of life [2,3]. TBI is particularly prevalent among disadvantaged populations, such as individuals who intersect with the criminal justice system (CJS) [4–8]. Prevalence rates of TBI among individuals who intersect with the CJS far exceed the rates found in the general population [5], with rates ranging from 25% to 86% among individuals who are incarcerated [7], 47% among those on probation [9], and 17% to 72% among youth [6]. Most recently, a meta-analysis published in 2023 found that the prevalence of TBI among individuals within the CJS was 46% [10].

Research has identified a relationship between having a history of TBI and experiencing adverse outcomes within the CJS. For example, a longitudinal study found that individuals completing sentences in federal correctional facilities who have a history of TBI were 39% more likely to incur serious disciplinary charges than those without a history of TBI [11]. Research has also demonstrated that a history of TBI is associated with an increased risk of reoffending upon release [12–15]. Unfortunately, health inequities experienced by individuals with TBI who intersect with the CJS also contribute to higher rates of chronic and infectious diseases, mental health challenges, substance use disorders, and serious psychological distress compared to the general population [16]. These health inequities are exacerbated by social determinants of health (SDoH), such as structural racism, that can limit healthcare access for groups that are disadvantaged, and lead to worse health outcomes for the high proportion of black, Indigenous, and people of colour who intersect with the CJS [17].

Clinical practice guidelines (CPGs) are “statements that include recommendations intended to optimise patient care that are informed by a systematic review of evidence and an assessment of the benefits and harms of alternative care options” [18]. However, despite their potential to enhance care, CPGs have been critiqued for dictating a one-size-fits-all approach to care [19] and lacking consideration for equity [20] or health inequities [21–23]. As such, existing CPGs may not be serving disadvantaged populations if they include recommendations that may not be applicable or beneficial to their needs. To the best of our knowledge, there has been no systematic review conducted to date that has examined the extent to which CPGs for TBI consider intersections with the CJS and the extent to which equity is considered in CPGs for CJS. This systematic review assesses the extent to which (1) intersections with the CJS is considered in CPGs for TBI; (2) TBI is considered in CPGs for CJS; and (3) equity is considered in CPGs for CJS.

## Methods

**Fig 1:**
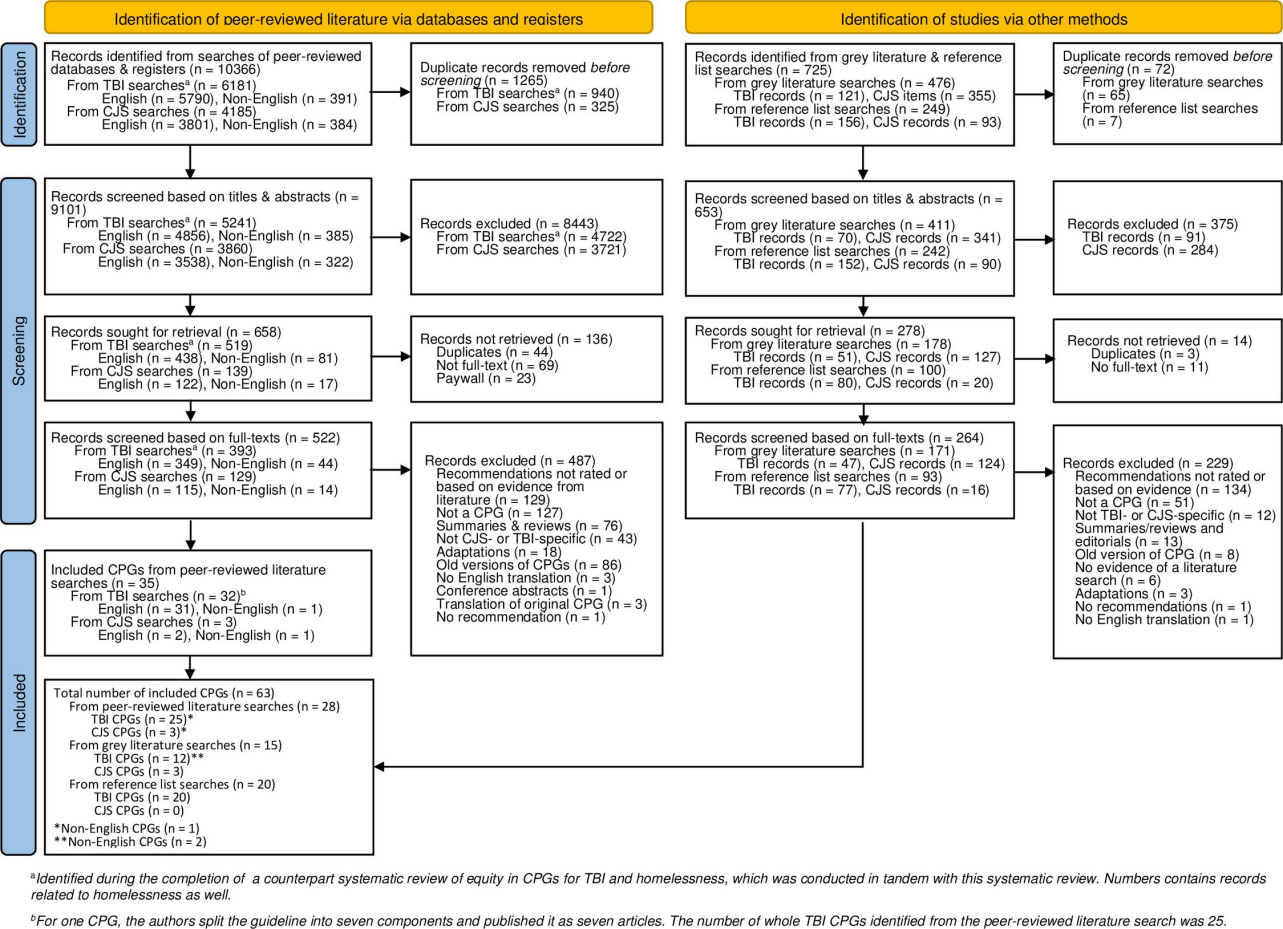
PRISMA flow chart. CJS, criminal justice system; CPG, clinical practice guideline; PRISMA, Preferred Reporting Items for Systematic Reviews and Meta Analyses; TBI, traumatic brain injury.

The protocol for this systematic review was registered on PROSPERO [CRD42022331499] and is an extension of our published systematic review assessing equity in CPGs for TBI and CPGs for individuals experiencing homelessness [24,25]. The reporting of the systematic review search strategy follows the Preferred Reporting Items for Systematic Reviews and Meta Analyses (PRISMA) extension for searching (PRISMA-S) [26,27] and the reporting of the systematic review followed the PRISMA Equity Extension [28] (please see **Fig 1**). A meta-analysis was not conducted, as the aim of the review was to specifically assess if and to what extent equity was considered; as such, combining and getting an overall effect estimate of the varying outcomes reported by CPGs was not necessary.

### Search strategy

CPGs were identified from (a) databases for peer-reviewed literature; (b) grey literature (i.e., targeted websites and Google Search); and (c) reference lists of eligible CPGs. Searches for CPGs for TBI and CPGs for CJS were conducted separately, as the process for identifying CPGs for TBI was completed as part of our published systematic review assessing CPGs for TBI and homelessness [24].

The CJS, in this review, encompasses 4 distinct facets in which direct involvement frequently occurs: policing (i.e., police interactions and arrests), courts (i.e., trials, including prosecution, adjudication, and sentencing), corrections (i.e., detention), and parole and probation [29–31]. Disadvantaged is a term used throughout this review to convey the lack of opportunities that ultimately place individuals in a disadvantaged position. While it is recognised that this term can be stigmatising, along with other terms such as marginalised or underserved, it was used to remain consistent with the language used by the Grading of Recommendations Assessment, Development, and Evaluation (GRADE) guideline series [32] that informed equity assessment for this review. Additionally, we recognise that the term “criminal legal system” is used more recently to refer to the criminal justice system; however, we decided to use “criminal justice system” in this paper to be consistent with the language currently used in CPGs.

#### Peer-reviewed literature

The search strategy was informed by a validated search for retrieving CPGs [33] and search strategies of scoping or systematic reviews of CPGs, TBI, and/or the CJS [24,25,34–36]. This search strategy was developed with an Information Specialist (JB) and team members with research and subject-matter expertise relevant to TBI and the CJS (VC, ZC, MJE). Additional details of the search strategy associated with each database for CPGs for CJS are outlined in **S1 Text**.

The overarching search strategy involved the search structure (a+b) OR (a+c) for text words and subject headings related to (a) CPG; (b) TBI; and (c) CJS. This search was conducted in 2 stages. The first stage was to identify CPGs for TBI (i.e., a+b), reported in a systematic review assessing equity in CPGs for TBI and CPGs for individuals experiencing homelessness [24]. The second stage was to identify CPGs related to CJS (i.e., a+c). For this stage, the following databases were searched: MEDLINE ALL (in Ovid, including Epub Ahead of Print, In-Process and Other Non-Indexed Citations, Ovid MEDLINE Daily), EMBASE and EMBASE Classic (Ovid), CINAHL (EBSCO), and APA PsycInfo (Ovid).

For both stages, no date or language limits were placed on the search strategies; however, where possible, we excluded animal studies and conference abstracts. Peer-reviewed literature searches for CPGs for TBI (stage 1) were conducted in November 2021 and in March 2023. Searches for CPGs for CJS (stage 2) were conducted in May 2022 and in March 2023.

#### Grey literature

The grey literature search strategy was informed by Goldin and colleagues’ methodology on applying systematic review search methods to grey literature [37] and used the same search structure of (a+b) or (a+c). Grey literature, defined as CPGs outside of the peer-reviewed literature, were identified from Google Search and targeted websites from Grey Matters: A Practical Tool for Searching Health-Related Grey Literature (hereafter referred to as “Grey Matters”) [38].

#### Reference list

Reference lists of scoping and systematic reviews and CPGs identified from the databases and the grey literature search were manually reviewed for CPGs that met inclusion criteria.

### Study selection

#### Eligibility criteria

CPGs for TBI and CPGs for CJS were screened separately. Only CPGs that met the eligibility criteria outlined in the PICAR statement [39] presented in **Table 1** were included in this review.

**Table 1:** PICAR statement for eligibility criteria.

	CPGs for TBI	CPGs for CJS involvement
**Inclusion criteria**		
**P: Population, Clinical Indicator(s), and Conditions(s)**	‐ TBI of any cause and severity of injury	‐ Individuals who intersect with any part of the CJS—policing, courts, corrections, and parole [29–31]
**I: Intervention(s)**	Any intervention	
**C: Comparator(s), Comparison(s), and (Key) Content**	Any comparator or comparison, no “key” CPG content is of interest (i.e., all content will be considered)	
**A: Attributes of CPG**	‐ CPGs are clearly evidence-based and demonstrate that a literature search was conducted ‐ Only the most recent version of the guideline will be included ‐ The entire full-text of the CPG must be attainable No limitations made based on additional characteristics such as country and year of publication, language, age of patients (e.g., pediatrics), population (e.g., veterans, athletes), setting (e.g., rehabilitation facilities, emergency departments, prisons), or the intended end-user (e.g., healthcare providers, patients)	
**R: Recommendation Characteristics and “Other” Considerations**	‐ CPG contains 1 or more evidence-based recommendation ‐ CPG uses a rating system to appraise the level of evidence behind the recommendation and outlines the appraisal tool	
**Exclusion criteria**		
**P: Population, Clinical Indicator(s), and Conditions(s)**	‐ CPGs focused on cognitive impairment without providing explicit recommendation(s) for TBI ‐ CPGs address general brain-injured populations without focusing on TBI (e.g., acquired brain injury)	‐ CPGs not explicitly stated as being for individuals who intersected with the CJS ‐ CPGs focused on non-criminal aspects of the legal system (e.g., civil legal system)
**I: Intervention(s)**	N/A	
**C: Comparator(s), Comparison(s), and (Key) Content**	N/A	
**A: Attributes of CPG**	‐ Editorials and summaries of guidelines ‐ Translations of guidelines ‐ Adaptations of existing guidelines for audiences other than intended end-user (e.g., guidelines for practitioners adapted for patients)	
**R: Recommendation Characteristics and “Other” Considerations**	‐ Strength of the recommendations in the CPGs are not rated ‐ Recommendations are not based on evidence from the literature	

CJS, criminal justice system; CPG, clinical practice guideline; N/A, not applicable; TBI, traumatic brain injury.

#### Peer-reviewed literature

EndNote X8 [40] was used for reference management and de-duplication, and Covidence [41] was used for de-duplication and study selection. Two independent reviewers (ZC, MJE, JMJ, or SH) screened all articles. Prior to formal title and abstract screening and full-text screening, a pilot screen of 20 articles and 10% of full-text articles, respectively, was conducted until a minimum 80% agreement was achieved between 2 independent reviewers. At the title and abstract screen, articles that focused on (a) the broader brain-injured population without specific mention of TBI or (b) non-criminal aspects of the legal system were included for the full-text screen to confirm whether they focused on the populations with TBI or those involved with the CJS. Scoping and systematic reviews of CPGs for TBI or CJS-involved individuals were also included and their reference lists were manually reviewed to identify additional CPGs not retrieved from the search. All non-English language CPGs were translated and assessed for eligibility using the English full-text translation, DeepL Translate, Google Translate, DocTranslate, or reviewers with knowledge of the language. CPGs that could not be translated were excluded and documented in the PRISMA flow chart. Discrepancies were resolved through consensus or by consulting a third reviewer (VC). The PRISMA flow chart [42] (**Fig 1**) illustrates the study selection process for peer-reviewed literature.

#### Grey literature

Two independent reviewers (ZC, MJE, JMJ, or SH) assessed all items on the Grey Matters checklist and the first 10 pages of Google Search to identify potentially relevant websites using the title and/or short text underneath the title. At the title and abstract screen, the executive summaries and/or table of contents were reviewed if an abstract was not available. Similar to the study selection of peer-reviewed literature, CPGs that focused on (a) the broader brain-injured population without specific mention of TBI; or (b) non-criminal aspects of the legal system were also considered for full-text review to confirm that they focused on populations with TBI or those involved with the CJS. Abstracts or executive summaries not in the English language were translated and assessed using identical procedures to the peer-reviewed literature screening. CPGs were then screened using the eligibility criteria outlined in **Table 1** for the full-text screen. The PRISMA flow chart [42] (**Fig 1**) illustrates the study selection process for grey literature.

#### Reference list

Similar to the grey literature screening, items identified from the reference list searches were added to an Excel file to generate a list of additional articles for review. Duplicates previously identified from the peer-reviewed and grey literature searches were removed prior to screening. The PRISMA flow chart [42] (**Fig 1**) illustrates the study selection process for the reference list search.

### Data extraction and synthesis

The method for data extraction and synthesis was adapted from Tannenbaum and colleagues’ review of CPGs for sex and gender considerations [43], which involved grouping or categorising CPGs into (1) text-positive and text-negative CPGs (see **S1 Data**); and (2) specific characteristics.

First, the content and reference lists of CPGs were assessed for (a) keywords for TBI and CJS involvement; and (b) content related to the definitions displayed in **Table 2**. CPGs were then categorised into text-positive and text-negative CPGs. Text-positive CPGs for TBI and CPGs for CJS were guidelines that contained at least one of the keywords for and content consistent with the definition of CJS involvement and TBI, respectively. Text-negative CPGs for TBI and CPGs for CJS were guidelines that, within the body of their text, did not contain keywords or content related to the definition of CJS involvement or TBI, respectively.

**Table 2:** Keywords and definitions for TBI and CJS involvement.

	Keywords describing TBI or CJS involvement or content consistent with the definition of TBI and parts of CJS involvement
**TBI**	Keywords: Brain injury or concussion or brain trauma or head injury or head trauma Definition [44]: An alteration in brain function, or other evidence of brain pathology, caused by an external force
**CJS involvement**	Keywords: Police or officer or arrest or offender or suspect or crime or criminal or justice trial or detention or probation or parole or legal or law or court or jail or prison or corrections or inmate or forensic Parts of CJS involvement [29–31]: a) Policing (e.g., involvement with police interactions and arrest procedures) b) Courts (e.g., involvement with prosecution and pretrial hearings, adjudication, and sentencing and sanctioning) c) Corrections (e.g., involvement with correctional facilities, including prisons and jails) d) Parole or probation (e.g., involvement with the parole or probation systems)

CJS, criminal justice system; TBI, traumatic brain injury.

Text-positive and text-negative CPGs were then further categorised by 2 independent reviewers (ZC, MJE, JMJ, or SH) using the categories and definitions outlined in **Table 3**. Data (i.e., quotes) that were used to categorise the guidelines were extracted. **S1 Data** presents the data extraction and synthesis for this systematic review.

**Table 3:** Tabulation addressing the 3 research objectives.

Objective	Definition and tabulation formula
1. Extent to which evidence about the CJS is integrated into CPGs for TBI	Proportion of text-positive guidelines for TBI = number of guidelines for TBI that was text-positive divided by the total number of guidelines for TBI included in the review
2. Extent to which evidence about TBI is integrated into CPGs for CJS	Proportion of text-positive guidelines for CJS = number of guidelines for CJS that was text-positive divided by the total number of guidelines for CJS included in the review
3. Extent to which equity is considered is CPGs	Proportion of CPGs that considered items from the equity lens and equity extension = number of guidelines that considered an equity item divided by the total number of guidelines included in the review

CPGs, clinical practice guidelines; CJS, criminal justice system; TBI, traumatic brain injury.

### Analysis

Narrative synthesis, informed by the Guidance on the Conduct of Narrative Synthesis in Systematic Reviews [45], was conducted. CPGs were grouped based on their characteristics (e.g., country, type of guideline, target audience), in addition to text-positive and text-negative categories. Tabulation (defined in **Table 3**), qualitative content analysis of text-positive CPGs, and quality appraisal were used to address the objectives of the review. Qualitative content analysis [46] were used to evaluate how evidence regarding TBI and CJS was integrated in CPGs for CJS and CPGs for TBI, respectively.

### Quality appraisal

Two independent reviewers (JMJ, MJE, or SH) completed quality appraisal using the equity lens from Dans and colleagues [21] and the equity extension from the GRADE working group [32,47–49] to assess equity considerations in included CPGs. Any technical, methodological, or supporting documents associated with the CPGs included in the review were retrieved to inform the quality appraisal process [39].

## Results

Sixty-three unique CPGs (4 of which were non-English language) met the eligibility criteria. Of these 63 CPGs, 6 were CPGs for CJS [50–55] and 57 were CPGs for TBI [56–120]. **Table 4** presents the characteristics of included CPGs.

**Table 4:** Characteristics of the CPGs.

**Characteristics of all CPGs (*N* = 63)**	***N* (%)**
**Year of publication** 2000–2004 [54,61,65,102] 2005–2009 [52,53,58,71,73,81,86,94,106] 2010–2014 [59,67,82,85,96,97,103,105,107,111] 2015–2019 [50,51,56,57,60,62,66,68,72,75–78,80,84,87,89,98,101,108–110] 2020–2023 [55,63,64,74,79,83,88,90–93,95,99,100,104,112–120]	4 (6.3) 9 (14.3) 10 (15.9) 22 (34.9) 18 (28.6)
**Country/countries of publication** Australia [55,80,81,85,102] Canada [63,78,83,84,87,88,96,97,111] France [65,66,75,76] Italy [62] New Zealand [86] Scotland [94] Spain [53] Taiwan [73] United Kingdom (England and Wales) [50,51,82,98] United States [52,54,58–61,64,67–69,71,72,74,77,79,89–93,95,99–101,104,106–110,115] Canada and the United States [56] Canada, United States, and Australia [112–114,116,117,119,120] Scandinavian countries (Denmark, Sweden, and Norway) [57,103,118] The Netherlands, Italy, Austria, Russia, Slovenia, Slovak Republic, Hungary, and Germany [105]	5 (7.9) 9 (14.3) 4 (6.3) 1 (1.6) 1 (1.6) 1 (1.6) 1 (1.6) 1 (1.6) 4 (6.3) 30 (47.6) 1 (1.6) 1 (1.6) 3 (4.8) 1 (1.6)
**CPGs for CJS (*N* = 6)**	***N* (%)**
**Focus of the guidelines** Mental health [50,52] Hepatitis viruses [53–55] Physical health [51]	2 (33.3) 3 (50.0) 1 (16.7)
**Age of target population** Adolescents—age not specified [52] Adolescents and adults—age not specified [54,55] Adults—age not specified [50,53] Adults ≥18 years [51]	1 (16.7) 2 (33.3) 2 (33.3) 1 (16.7)
**Target CJS population**^**a**^ Individuals detained within the correctional system [51–55] Correctional workers [54] Not specified [50]	5 (83.3) 1 (16.7) 1 (16.7)
**Target audience**^**a**^ Healthcare professionals [50–55] Allied health professionals [50–52,55] Patients in the CJS and their families [53]	6 (100.0) 4 (66.7) 1 (16.7)
**Type of CJS intersection mentioned in guidelines**^**a**^ Policing [50] Court [50] Corrections [50–55] Parole [50]	1 (16.7) 1 (16.7) 6 (100.0) 1 (16.7)
**CPGs for TBI (*N* = 57)**	***N* (%)**
**Focus of the guidelines**^**a**^ Assessment/evaluation [59,60,62,64,67,69,74,78,80,82–84,87,90,97,104,115] Management [56–59,61–68,70–73,76–86,88,89,91,92,94,95,98,100–108,111,115,118,121] Treatment [60,69,74,77,87,93,108] Diagnosis or screening [68,77,81,86,88,89,91,95,98,99,102,109,110] Rehabilitation [80,86,90,100,108,112–114,116,117,119,120] Prevention [59,75] Return to activity [59,63,77,96]	17 (29.8) 45 (78.9) 6 (10.5) 13 (22.8) 6 (10.5) 2 (3.5) 4 (7.0)
**Injury severity** Mild/concussion [59,63,64,67,68,77–79,81,83,87–90,92,96,99–101,105,107,111,115,118] Severe [56,60,61,65,66,69,72,73,104] Mild or moderate [57,103] Moderate or severe [80,84,112–114,116,117,119,120] All severity [85,86,91,95,97,109] Not specified [58,62,71,74–76,82,93,94,98,102,106,108,110]	24 (42.1) 8 (14.0) 2 (3.5) 3 (5.3) 6 (10.5) 14 (24.6)
**Age of target population** Children <16 years [62,91] Children <18 years [57,72,77,80,89] Children ages 5–18 [88] Children and adolescents—age not specified [63,79,92,104] Children and adults ≥8 years [90] Adults—age not specified [60,61,64,69,75,76,84,98,100,102,115] Adults ≥16 years [56,81,85,95] Adults ≥18 years [78,87,101,103,112–114,116–120] Adults 18–65 years [97] All ages [58,59,65,66,71,73,82,83,86,94,105] Target age not specified [67,68,74,93,96,99,106–111]	2 (3.5) 5 (8.8) 1 (1.8) 4 (7.0) 1 (1.8) 10 (17.6) 4 (7.0) 6 (10.5) 1 (1.8) 11 (19.3) 12 (21.1)
**Target population**^**a**^ Not specified [56–69,71–79,82,84,86–89,92–94,96–99,103–110,112–120] Sports/athletes [59,67,68] Military [100,101] Injured workers [111] At-risk for speech, language, and/or swallowing disorders [80] TBI from road traffic accident [102] Other [81,83,85,90,91,95]	46 (80.7) 3 (5.3) 2 (3.5) 1 (1.8) 1 (1.8) 1 (1.8) 6 (10.5)
**Target audience**^**a**^ Healthcare professionals [56,58–61,63–69,71–80,82–84,86–89,91–102,104–106,108–114,116–120] Clinicians/physicians [57,62,81,85,103] Allied health professionals [78,80,87,100,108,112–114,116,117,119,120] Nurses [78,87,101,107,108] Physiotherapist [90] Emergency medical service provider [58,115] Athletic trainer/sports organizations [59] Families/caregivers/persons with TBI [63,67,75,82,86,94,97,102,112–114,116,117,119,120] Employers [97]	49 (86.0) 5 (8.8) 6 (10.5) 5 (8.8) 1 (1.8) 2 (3.5) 1 (1.8) 9 (15.8) 1 (1.8)
**Facets of CJS intersection from text-positive CPGs for TBI**^**a**^ Policing [81,82,84,90,102 and 118] Corrections [75,76,86,106,115] Parole [84] Other [57,76,86,97,112–114,116,117,119 and 120]^b^	6 (10.5) 5 (8.8) 1 (1.8) 5 (8.8)

^a^Each CPG may target more than one focus, population, CJS intersection, or audience; thus, the total *N* and % will not equal to 100%.

^b^Other facets of CJS intersection include involvement with legal problems and/or criminal behaviour, legal investigations, and unspecified medical-legal or forensic contexts.

CPG, clinical practice guidelines; CJS, criminal justice system; TBI, traumatic brain injury.

### Inclusion of CJS information in CPGs for TBI

Fourteen CPGs for TBI (24.6%) were text-positive for CJS-related keywords or content [57,75,76,81,82,84,86,90,97,102,106,112–120]; one CPG provided an evidence-based recommendation for individuals with CJS involvement (i.e., category 1) [97], 7 acknowledged or made reference to data regarding individuals with CJS involvement without recommendations (i.e., category 2) [57,81,84,86,90,112–117,119,120], and 6 mentioned individuals with CJS involvement without providing context related to the literature or recommendations (i.e., category 3) [75,76,82,102,106,118].

Three CPGs mentioned CJS-related keywords in the context of TBI evaluation in the CJS [86,97,102], one of which provided a concrete recommendation regarding vocational evaluation in the forensic context, noting that evaluators must consider medical-legal contexts during vocational evaluation, as they can influence the validity, completion, and reporting of evaluation findings [97]. One incorporated history of arrests in a checklist for behavioural affective symptoms associated with TBI [102] while the other identified prison inmates as a group who may not have received an initial assessment despite sustaining a TBI [86] but did not provide further recommendations.

Three CPGs reported on the role of CJS staff in increasing awareness about TBI, but did not provide recommendations for individuals with TBI who intersect with the CJS [81,82,84]. These CPGs highlighted the role of police officers in encouraging patients to immediately seek medical advice regarding TBI that they or others have sustained, regardless of injury severity [82], and ensuring that patients with TBI understand the risks of not being transported to the hospital following a head injury [81]. One CPG highlighted the need for TBI rehabilitation programs to increase awareness regarding TBI through information and education activities regarding the challenges and needs of individuals with TBI. This CPG listed police and parole officers under groups most likely to encounter individuals with TBI [84].

Three CPGs mentioned CJS keywords when associating TBI with criminal behaviour [86,112–117,119,120]. One CPG noted that symptoms of TBI (e.g., cognitive impairments, functional disability, and difficulties regulating emotions) can hinder return-to-work and lead to impairments in interpreting social situations, thereby increasing individuals’ susceptibility to criminal behaviour [112–114,116,117,119,120]. Another CPG acknowledged the positive correlation between TBI and incarceration among Māori people [86]. The same CPG also noted the presence of sexually aberrant behaviours following TBI that can lead to sexual offences against medical staff, patients, and their families [86].

Two CPGs integrated CJS keywords in return-to-work considerations [81,86], specifically police, criminal record, litigation, and legal requirements when helping an individual return to work [81]. For example, the CPG noted the need for the person with TBI and their caregivers to be informed regarding legal requirements for driving (e.g., log books, licencing procedures, and license revocation procedures) and fitness-to-drive post-injury [81,86].

Five CPGs integrated CJS-related information when reporting on guideline development [75,76,86,106,118]. One CPG listed police associations as a target user of the CPG [118], while 2 mentioned lawyers and physicians working in prison in the groups that contributed to the development recommendations [75,76]. One CPG included an explanation of the guideline development process and listed “effective identification of TBI in prisons” as part of the agreed-upon list of topics to be covered by the guideline [86]. One reported not including literature on aggression among prison populations, as it was too difficult to generalise to the larger population of individuals with brain injury [106].

**Fig 2:**
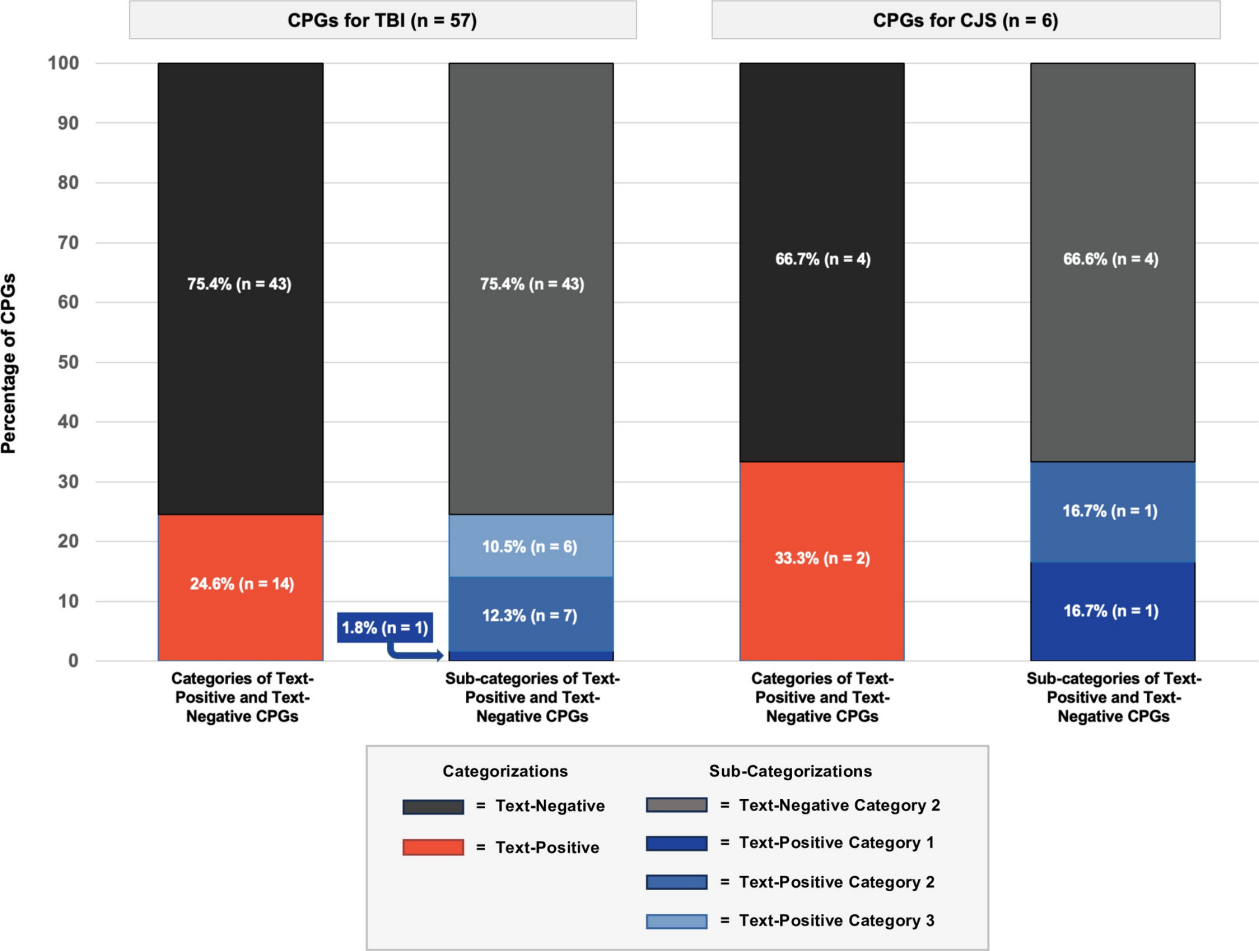
Proportion of text-positive and text-negative CPGs. CJS, criminal justice system; CPG, clinical practice guideline; TBI, traumatic brain injury.

Some CPGs included CJS-specific keywords in contexts not relevant to individuals with TBI who intersect with the CJS. Such contexts include child abuse reporting [57], concussions and physical therapy for police officers [90], medico-legal implications or ramifications of appropriate TBI evaluations [85], and the utilisation of pharmacologic treatments [76]. The proportion of text-positive and text-negative CPGs for TBI for each category is summarised in **Fig 2**.

### Inclusion of TBI information in CPGs for CJS

Two CPGs for CJS (33.3%) were text-positive for TBI keywords [50,51]. The National Institute for Health and Care Excellence (NICE) 2017 guideline for mental health in the criminal justice system referenced epidemiological data regarding individuals with TBI without recommendations (category 2) [50], specifically noting the prevalence of TBI among individuals in prison compared to the general population. The NICE 2016 guideline for physical health of people in prison provided evidence-based recommendations for individuals with TBI (i.e., category 1), [51] to consider head injury in health assessments for all individuals in prison. Specifically, at first reception into prison, healthcare professionals should assess physical injuries (including head injuries) and document any treatments received. Further questions concerning the frequency of head injuries and/or loss of consciousness (LOC), the duration of the LOC, and difficulties with memory or concentration should then be asked during the second-stage health assessments [51]. The remaining 4 CPGs for CJS were text-negative and included no keywords or content specific to TBI within the body of the guidelines or their reference lists. The proportion of text-positive and text-negative CPGs for CJS for each category is summarised in **Fig 2**.

### Quality appraisal

CPGs for CJS were often cognizant of equity issues as these guidelines were developed specifically for individuals intersecting with the CJS, a disadvantaged population. All CPGs for CJS offered distinct recommendations for those who are disadvantaged [50–55]; 4 (66.7%) noted differences in disease epidemiology between privileged versus disadvantaged populations [50,52,54,55], and 5 (83.3%) addressed the burden of disease on disadvantaged groups [50,52–55], provided solutions to barriers that hinder the implementation of recommendations in these populations [50–52,54,55], and evaluated data on cost, resource use, impact on equity, acceptability, and viability of interventions for populations that were disadvantaged [50–54].

A minority of CPGs for CJS reported involving disadvantaged groups in the development of the guideline. Two CPGs (33.3%) involved disadvantaged groups in their guideline development group and consulted them in determining the value of interventions and their outcomes [50,51]. None reported considering representatives from pertinent groups that were disadvantaged when ascertaining target audiences of the guidelines or recruiting methodologists or voting panel chairs who were familiar with equity issues.

Equity considerations in searching, synthesising, and reporting evidence on disadvantaged groups were often present in CPGs for CJS. Five CPGs (83.3%) reported searching for evidence specific to populations that were disadvantaged [50–54], and 2 (33.3%) explored databases for intervention outcomes that were critical to disadvantaged populations, addressed evidence from non-health-related disciplines that consider disadvantaged populations, and made considerations for equity when specifying the evidence eligibility criteria [50,51]. Three CPGs were published after the development of the PRISMA-equity statement [50,51,55]; however, none reported following the PRISMA-equity statement when reporting findings of systematic reviews and including good practice statements that address equity issues.

The majority of CPGs for CJS provided recommendations related to the implementation of the guidelines. Two offered specific tools for implementing the guidelines [50,51]; 5 provided clarifications for recommendations to ensure that the guideline is properly implemented [50–54], and offered strategies to monitor groups according to PROGRESS-plus elements [50–52,54,55]. One had recommendations related to monitoring the use of the guideline among disadvantaged groups [50].

Four CPGs for CJS were devoted entirely to singular health issues (hepatitis viruses [53–55] and mental health [50]). CPGs for CJS included considerations for culture [50,52,54,55], race and/or ethnicity [50–52,54,55], geographic location and proximity to medical care [50,51], financial status [51,52], disability [50,51,54], as well as sexual identity, sex, and gender [50–52,54]. However, these considerations were often only present in the reporting or synthesis of evidence and rarely in the guidelines’ list of recommendations. Specifically, only 2 of the 6 CPGs for CJS included evidence-based recommendations that highlighted disability [50,51] and only 4 highlighted gender or sex [50–52,54] and race or ethnicity [50,52,54,55].

**Fig 3:**
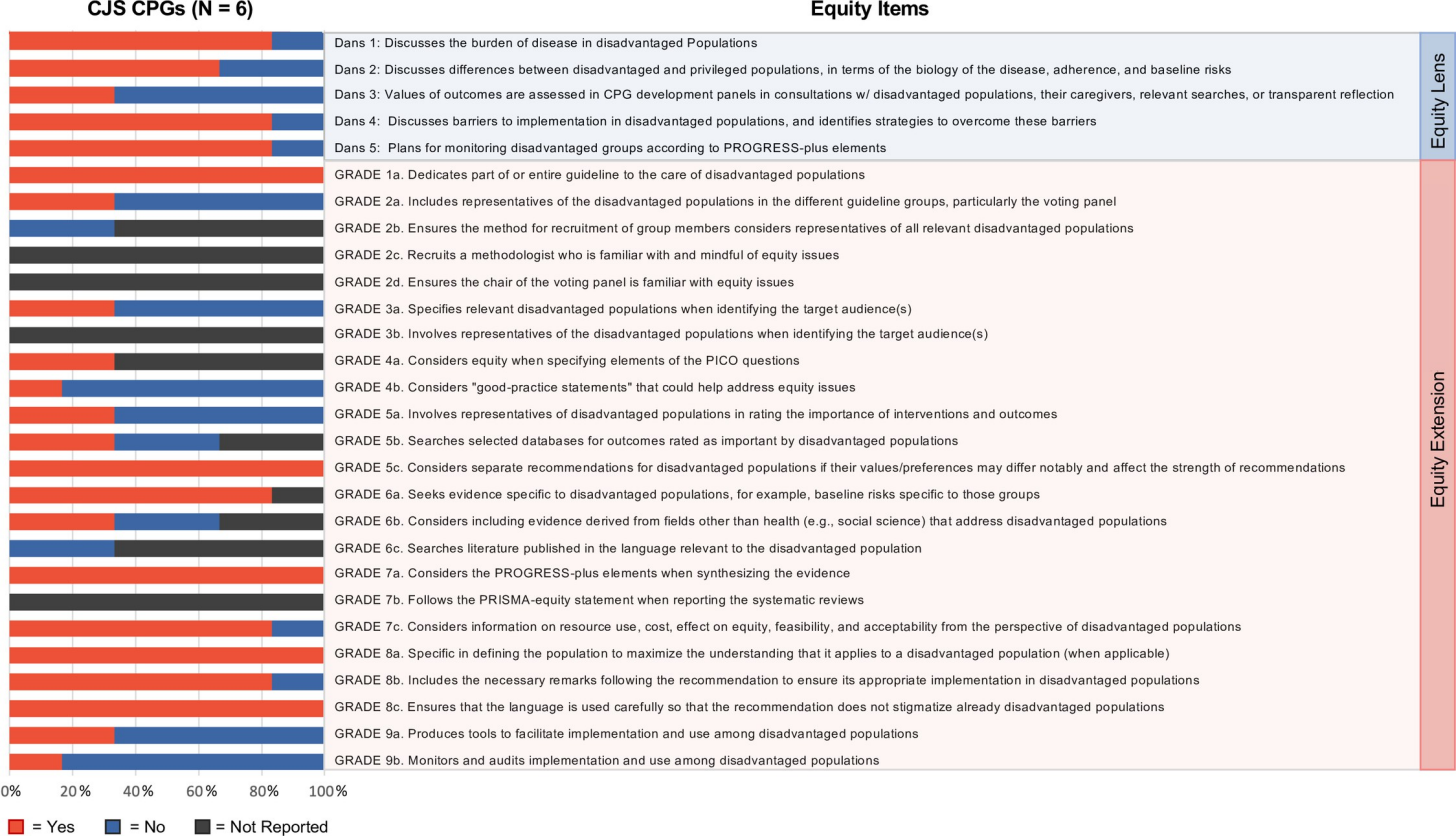
Summary of equity considerations in CPGs for CJS. CJS, criminal justice system; CPG, clinical practice guideline.

Quality appraisal results of CPGs for CJS are presented in **S2 Data** and **Fig 3**. Please refer to a separate systematic review on assessing equity for CPGs for TBI and homelessness for the equity assessment of CPGs for TBI [24].

## Discussion

To the best of our knowledge, this is the first systematic review to explore the extent to which CPGs for TBI consider CJS intersection or involvement and, likewise, the degree to which CPGs for CJS consider TBI. This review also evaluated equity considerations in CPGs for CJS. Findings show that evidence-based recommendations for individuals with TBI who intersect with the CJS and equity considerations are lacking in CPGs for TBI and CPGs for CJS. We identified the following opportunities to advance equity in healthcare for individuals with TBI who intersect with the CJS: (1) conduct research with disadvantaged groups; (2) investigate TBI screening in all parts of the CJS, and (3) utilise equity assessment tools in guideline development.

First, there is an urgent need to conduct research with disadvantaged groups to build the evidence base on the intersections of TBI, CJS, health equity, and SDoH. Our findings showed the lack of specific evidence-based guidance regarding TBI care for individuals who intersect with the CJS. While a quarter of CPGs for TBI included CJS keywords, only one provided a concrete evidence-based recommendation regarding vocational evaluation for individuals who intersect with the CJS [97]. In contrast, only two of the CPGs for CJS included were text-positive for TBI, and only one of the two provided a specific recommendation to consider TBI when assessing the health of individuals in prison [51]. Specifically, individuals should be asked if they ever suffered a head injury or lost consciousness. Follow-up questions should include the number of times they suffered a head injury or lost consciousness, how long they were unconscious, and whether or not they have challenges with memory or concentration [51]. However, no specific instruments or tools to screen for a TBI were recommended. Policing and corrections were the predominant focus in CPGs for TBI and CPGs for CJS, with the courts, parole, and probation systems being largely neglected. It is also worth noting that 4 out of 7 CPGs that focused on policing were focused on police officers as patients themselves and not necessarily police interactions with individuals with TBI. The largely singular focus of the CPGs is likely due to the overwhelming focus of research on the CJS on persons who experience incarceration [122]. However, other parts of the CJS, such as parole, probation and courts, require equal attention, given the overrepresentation of TBI among persons on probation [9], the lower rates of successful probation completion among those with TBI, and the difficulties experienced by individuals with TBI in comprehending legal language [123]. More research focusing on, and with individuals who intersect with all parts of the CJS, is needed to build the evidence base and advance care for this population.

Second, this review identified an opportunity to investigate TBI screening in all parts of the CJS. For decades, public health organisations such as the Centers for Disease Control and Prevention have recognised the high prevalence of TBI in individuals intersecting with the CJS as a public health problem [124]. However, to date, TBI screening is inconsistently implemented in the CJS [125–127] due to a lack of funding and staff, poor awareness of TBI, and high rates of turnover among individuals in prison [126]. The findings of this systematic review echo this contrast. While a significant portion of TBI guidelines recognised intersections with the CJS as an important consideration, screening for TBI was not mentioned; only 1 out of 6 CJS CPGs recommended inquiring about head injuries when assessing the physical health of individuals in prison [51]. Importantly, several studies have reported on the potential benefits of screening for TBI in the CJS. A scoping review on rehabilitation programs for individuals with TBI who intersect with the CJS found that TBI screening is a crucial first step in the identification of unmet needs and the development of individualised intervention plans for this group [127]. Another study found that the aggressive and violent behaviours often exhibited by individuals with TBI are correlated with recidivism, and TBI screening in correctional facilities can be used to identify individuals at risk for reoffending, advise inmate behavioural management, and improve their safety [128]. These findings acknowledge the potential of TBI screening in supporting individuals with TBI. However, despite these potential benefits, we also recognise that screening for TBI may come with the risk of being identified as someone with a potential cognitive disability, which could put persons in the CJS at further risk of victimisation. As such, there is a clear need to further explore and understand the best way to incorporate screening for TBI in all parts of the CJS. Considerations for screening, including duration of screening, recommended tools and resources, procedures and supports following screening, and communication of screening results between different levels of the CJS and the community need to be explored through future research. Such information will also inform the feasibility, processes, and implications of screening for TBI in all parts of the CJS [127].

Lastly, through our assessment of equity, we identified a need to utilise equity assessment tools in guideline development and to incorporate considerations for disadvantaged groups into the evidence-based recommendations. CPGs for CJS lacked good practice statements and did not report using the PRISMA equity statement even though half were published after the statement’s publication in 2012. Considerations for race or ethnicity, financial status, and disability were largely absent from the recommendations. These considerations are important to include, as SDoH such as sex, gender, race, disability, and financial status can implicate the relevance and efficacy of the recommended interventions. Incorporating these equity best practices in the formulation of recommendations and using equity assessment tools in the development of CPGs are necessary first steps to reduce healthcare disparities between privileged and disadvantaged populations. Ultimately, the findings from this review provide a foundation to address equitable healthcare for individuals with TBI who intersect with the CJS.

While our review addressed an important gap in the literature regarding TBI, CJS, and equity considerations in CPGs, we recognise the following limitations. First, we did not systematically search for research on individuals with TBI who intersect with the CJS, outside of CPGs. As such, we are unable to comment on evidence that is not integrated into existing CPGs. Second, while we did not place restrictions on language or country in our search, our searches were conducted in English language. As such, we may have missed other non-English language CPGs in this review. Finally, although our eligibility criteria were selected in accordance with guideline development best practices [129], selecting only CPGs that rated the strength of their recommendations may have resulted in the exclusion of CPGs from certain lower-income countries, where rating the level of evidence or recommendations is a less prevalent or not commonly reported practice [130,131].

Despite these limitations, our systematic review is strengthened by its application of the externally peer-reviewed and published protocol of a systematic review that was conducted in tandem with this review [25], as well as the rigorous search strategy. Validated search filters for CPGs [33] and methodological guides to searching grey literature [37,39] were used to increase transparency and replicability. Additionally, our search strategy for searching grey literature was a core strength, particularly given the enhanced precision that has been shown to be associated with searches beyond bibliographic databases [33]. Furthermore, this systematic review assessed numerous key facets of the CJS, thus providing a comprehensive overview of CJS intersection. This was important because although research on individuals in correctional facilities is ample, the remaining facets of the CJS are largely neglected. As such, our systematic review highlights the gaps in the literature regarding the court, sentencing, and parole systems [132]. Lastly, through the identification of text-positive CPGs, we have established a collection of CPGs for TBI that address the needs of those within the CJS and CPGs for CJS that address individuals with TBI, which serve as a foundation for individuals providing TBI care in forensic settings.

Collectively, this systematic review highlights a lack of published guidance for the care of individuals with TBI who intersect with the CJS and an overwhelming need to recognise healthcare inequities for individuals intersecting with the CJS and take meaningful action to address current gaps in guidance for care delivery. Critical next steps in advancing equity and healthcare for this disadvantaged group include continuing to grow an evidence base for the intersection of TBI and CJS care, addressing the vulnerabilities stemming from brain injury that are unique to those who intersect with the CJS within our approaches to care, exploring opportunities to screen for TBI across all facets of the CJS to understand the impacts of brain injury among those who intersect with the CJS, and utilizing equity assessment tools in the development and implementation of CPGs. Furthermore, it is important that CPGs make considerations for the differing needs of distinct populations and address relevant SDoH, such as sex, gender, and race, as the healthcare needs, effectiveness of clinical recommendations, and structural barriers for different groups can vary substantially. Achieving these goals will be a step forward in delivering high-quality care for disadvantaged populations experiencing TBI.

## Supporting information

S1 Table PRISMA checklists. (PDF)

S1 Text Search strategy. (PDF)

S1 Data Data extraction and synthesis. (PDF)

S2 Data Quality appraisal tables. (PDF)

## Abbreviations

CJS: criminal justice system
CPG: clinical practice guideline
GRADE: Grading of Recommendations Assessment, Development, and Evaluation
LOC: loss of consciousness
NICE: National Institute for Health and Care Excellence
PRISMA: Preferred Reporting Items for Systematic Reviews and Meta Analyses
SDoH: social determinants of health
TBI: traumatic brain injury
