# Supplementary material for: Equity considerations in clinical practice guidelines for traumatic brain injury and the criminal justice system: A systematic review
Source: PLoS Med. 2024 Aug 12;21(8):e1004418. doi: 10.1371/journal.pmed.1004418 (PMC11319042; doi:10.1371/journal.pmed.1004418)
Supplement: S1 Table — (PDF) [file pmed.1004418.s001.pdf]

S1 Table. PRISMA Checklists

Table A: PRISMA-S Checklist

Section/topic	#	Checklist item	Location(s) Reported
**INFORMATION SOURCES AND METHODS**			
Database name	1	Name each individual database searched, stating the platform for each.	Methods > Search Strategy > Peer-Reviewed Literature (Paragraph 2), and S1 Text (Search Strategy)
Multi-database searching	2	If databases were searched simultaneously on a single platform, state the name of the platform, listing all of the databases searched.	Methods > Search Strategy > Peer-Reviewed Literature (Paragraph 2), and S1 Text (Search Strategy)
Study registries	3	List any study registries searched.	Methods > Search Strategy > Peer-Reviewed Literature (Paragraph 2), and S1 Text (Search Strategy)
Online resources and browsing	4	Describe any online or print source purposefully searched or browsed (e.g., tables of contents, print conference proceedings, web sites), and how this was done.	Methods > Search Strategy > Grey literature and Reference List, and S1 Text (Search Strategy)
Citation searching	5	Indicate whether cited references or citing references were examined, and describe any methods used for locating cited/citing references (e.g., browsing reference lists, using a citation index, setting up email alerts for references citing included studies).	Methods > Search Strategy > Reference List
Contacts	6	Indicate whether additional studies or data were sought by contacting authors, experts, manufacturers, or others.	N/A
Other methods	7	Describe any additional information sources or search methods used.	N/A
**SEARCH STRATEGIES**			

Full search strategies	8	Include the search strategies for each database and information source, copied and pasted exactly as run.	S1 Text (Search Strategy)
Limits and restrictions	9	Specify that no limits were used, or describe any limits or restrictions applied to a search (e.g., date or time period, language, study design) and provide justification for their use.	Methods > Search Strategy > Peer-Reviewed Literature (Paragraph 3)
Search filters	10	Indicate whether published search filters were used (as originally designed or modified), and if so, cite the filter(s) used.	S1 Text (Search Strategy)
Prior work	11	Indicate when search strategies from other literature reviews were adapted or reused for a substantive part or all of the search, citing the previous review(s).	Methods > Search Strategy > Peer-Reviewed Literature (Paragraph 1), and S1 Text (Search Strategy)
Updates	12	Report the methods used to update the search(es) (e.g., rerunning searches, email alerts).	S1 Text (Search Strategy)
Dates of searches	13	For each search strategy, provide the date when the last search occurred.	S1 Text (Search Strategy)
**PEER REVIEW**			
Peer review	14	Describe any search peer review process.	Methods > Study Selection > Peer-Reviewed Literature, Grey Literature, and Reference List, and S1 Text (Search Strategy)
**MANAGING RECORDS**			
Total Records	15	Document the total number of records identified from each database and other information sources.	PRISMA Flow-Chart (Figure 1)
Deduplication	16	Describe the processes and any software used to deduplicate records from multiple database searches and other information sources.	Methods > Study Selection > Peer-Reviewed Literature, Grey Literature, and Reference List

This table was obtained from the PRISMA-S Statement for Reporting Literature Searches in Systematic Reviews[1]

Table B: PRISMA Equity Checklist

Checklist of Items for Reporting Equity-Focused Systematic Reviews				
**Section**	**Item**	**Standard PRISMA Item**	**Extension for Equity-Focused Reviews**	**Pg #**
**Title**				
**Title**	1	Identify the report as a systematic review, meta-analysis, or both.	Identify equity as a focus of the review, if relevant, using the term equity	Title page
**Abstract**				
**Structured summary**	2	Provide a structured summary including, as applicable: background; objectives; data sources; study eligibility criteria, participants, and interventions; study appraisal and synthesis methods; results; limitations; conclusions and implications of key findings; systematic review registration number.	State research question(s) related to health equity.	Abstract > Background
	2A		Present results of health equity analyses (e.g. subgroup analyses or meta-regression).	Abstract > Methods and findings
	2B		Describe extent and limits of applicability to disadvantaged populations of interest.	Abstract > Methods and findings
**Introduction**				
**Rationale**	3	Describe the rationale for the review in the context of what is already known.	Describe assumptions about mechanism(s) by which the intervention is assumed to have an impact on health equity.	N/A
	3A		Provide the logic model/analytical framework, if done, to show the pathways through which the intervention is assumed to affect health equity and how it was developed.	N/A
**Objectives**	4	Provide an explicit statement of questions being addressed with reference to participants, interventions, comparisons, outcomes, and study design (PICOS).	Describe how disadvantage was defined if used as criterion in the review (e.g. for selecting studies, conducting analyses or judging applicability).	Introduction (Paragraphs 1 and 2)
	4A		State the research questions being addressed with reference to health equity	Introduction (Paragraph 3)
**Methods**				
**Protocol and registration**	5	Indicate if a review protocol exists, if and where it can be accessed (e.g., Web address), and, if available,		Methods (Paragraph 1)

		provide registration information including registration number.		
**Eligibility criteria**	6	Specify study characteristics (e.g., PICOS, length of follow-up) and report characteristics (e.g., years considered, language, publication status) used as criteria for eligibility, giving rationale.	Describe the rationale for including particular study designs related to equity research questions.	N/A
	6A		Describe the rationale for including the outcomes - e.g. how these are relevant to reducing inequity.	N/A
**Information sources**	7	Describe all information sources (e.g., databases with dates of coverage, contact with study authors to identify additional studies) in the search and date last searched.	Describe information sources (e.g. health, non-health, and grey literature sources) that were searched that are of specific relevance to address the equity questions of the review.	Methods > Peer-Reviewed Literature (Paragraph 2), Grey Literature, and Reference list
**Search**	8	Present full electronic search strategy for at least one database, including any limits used, such that it could be repeated.	Describe the broad search strategy and terms used to address equity questions of the review.	N/A
**Study selection**	9	State the process for selecting studies (i.e., screening, eligibility, included in systematic review, and, if applicable, included in the meta-analysis).		Methods > Study Selection
**Data collection process**	10	Describe method of data extraction from reports (e.g., piloted forms, independently, in duplicate) and any processes for obtaining and confirming data from investigators.		Methods > Data Extraction and Synthesis
**Data items**	11	List and define all variables for which data were sought (e.g., PICOS, funding sources) and any assumptions and simplifications made.	List and define data items related to equity, where such data were sought (e.g. using PROGRESS-Plus or other criteria, context).	Table 3, S1 Data, and S2 Data
**Risk of bias in individual studies**	12	Describe methods used for assessing risk of bias of individual studies (including specification of whether this was done at the study or outcome level), and how this information is to be used in any data synthesis.		N/A
**Summary measures**	13	State the principal summary measures (e.g., risk ratio, difference in means).		Table 3

**Synthesis of results**	14	Describe the methods of handling data and combining results of studies, if done, including measures of consistency (e.g., I^2) for each meta-analysis.	Describe methods of synthesizing findings on health inequities (e.g. presenting both relative and absolute differences between groups).	Table 3, and Methods > Quality Appraisal
**Risk of bias across studies**	15	15. Specify any assessment of risk of bias that may affect the cumulative evidence (e.g., publication bias, selective reporting within studies).		N/A
**Additional analyses**	16	Describe methods of additional analyses (e.g., sensitivity or subgroup analyses, meta-regression), if done, indicating which were pre-specified.	Describe methods of additional synthesis approaches related to equity questions, if done, indicating which were pre-specified	N/A
**Results**				
**Study selection**	17	Give numbers of studies screened, assessed for eligibility, and included in the review, with reasons for exclusions at each stage, ideally with a flow diagram.		PRISMA Flow-Chart (Figure 1)
**Study characteristics**	18	For each study, present characteristics for which data were extracted (e.g., study size, PICOS, follow-up period) and provide the citations.	Present the population characteristics that relate to the equity questions across the relevant PROGRESS-Plus or other factors of interest.	S1 Data, and S2 Data
**Risk of bias within studies**	19	Present data on risk of bias of each study and, if available, any outcome level assessment (see item 12).		N/A
**Results of individual studies**	20	For all outcomes considered (benefits or harms), present, for each study: (a) simple summary data for each intervention group (b) effect estimates and confidence intervals, ideally with a forest plot.	a	N/A
**Synthesis of results**	21	Present results of each meta-analysis done, including confidence intervals and measures of consistency.	Present the results of synthesizing findings on inequities (see 14).	Results > Inclusion of CJS information in CPGs for TBI, inclusion of TBI information in CPGs for CJS, and Quality Appraisal, Figure 3, S1 Data, and S2 Data
**Risk of bias across studies**	22	Present results of any assessment of risk of bias across studies (see Item 15).		N/A

**Additional analysis**	23	Give results of additional analyses, if done (e.g., sensitivity or subgroup analyses, meta-regression [see Item 16]).	Give the results of additional synthesis approaches related to equity objectives, if done, (see 16).	N/A
**Discussion**				
**Summary of evidence**	24	Summarize the main findings including the strength of evidence for each main outcome; consider their relevance to key groups (e.g., healthcare providers, users, and policy makers).		Discussion (Paragraphs 1 to 5)
**Limitations**	25	Discuss limitations at study and outcome level (e.g., risk of bias), and at review-level (e.g., incomplete retrieval of identified research, reporting bias).		Strengths and Limitations (Paragraph 1)
**Conclusions**	26	Provide a general interpretation of the results in the context of other evidence, and implications for future research.	Present extent and limits of applicability to disadvantaged populations of interest and describe the evidence and logic underlying those judgments.	Strengths and Limitations (Last Paragraph)
	26A		Provide implications for research, practice or policy related to equity where relevant (e.g. types of research needed to address unanswered questions).	Strengths and Limitations (Last Paragraph)
**Funding**				
**Funding**	27	Describe sources of funding for the systematic review and other support (e.g., supply of data); role of funders for the systematic review.		Acknowledgments

This table was obtained from the PRISMA-Equity 2012 Extension: Reporting Guidelines for Systematic Reviews with a Focus on Health Equity[2]

[1] PRISMA-S: An Extension to the PRISMA Statement for Reporting Literature Searches in Systematic Reviews Rethlefsen ML, Kirtley S, Waffenschmidt S, Ayala AP, Moher D, Page MJ, Koffel JB, PRISMA-S Group.

[2] Welch V, Petticrew M, Tugwell P, Moher D, O'Neill J, Waters E, White H, and the PRISMA-Equity Bellagio Group. (2012) PRISMA-Equity 2012 Extension: Reporting Guidelines for Systematic Reviews with a Focus on Health Equity. PLoS Med 9(10): e1001333. doi:10.1371/journal.pmed.1001333
