# Supplementary material for: Equity considerations in clinical practice guidelines for traumatic brain injury and the criminal justice system: A systematic review
Source: PLoS Med. 2024 Aug 12;21(8):e1004418. doi: 10.1371/journal.pmed.1004418 (PMC11319042; doi:10.1371/journal.pmed.1004418)
Supplement: S1 Text — (PDF) [file pmed.1004418.s002.pdf]

S1 Text. Search Strategy

Peer-Reviewed Literature Search Strategy

Database: Ovid MEDLINE(R) ALL <1946 to March 15, 2023>
Platform: Ovid
Date searched: 2023-03-16

1 exp PRISONS/
2 exp PRISONERS/
3 exp CRIMINALS/
4 Criminal Law/ or Jurisprudence/
5 Judicial Role/
6 (jurisprudenc* or ligitat*).tw,kf.
7 (legal adj (system? or servic*)).tw,kf.
8 (prisoner* or prison? or imprison*).tw,kf.
9 (inmate* or convict* or criminal* or offender?).tw,kf.
10 (correctional adj2 (setting? or service? or units or unit or facility or facilities or institution* or centre* or center*)).tw,kf.
11 (penal adj2 (setting? or service? or units or unit or facility or facilities or institution* or centre* or center*)).tw,kf.
12 (jail* or penitentiar* or gaol*).tw,kf.
13 incarcerat*.tw,kf.
14 (detain* or detention?).tw,kf.
15 parole?.tw,kf.
16 probation*.tw,kf.
17 felon*.tw,kf.
18 Police/
19 (police or policing).tw,kf.
20 law enforce*.tw,kf.
21 forensic*.tw,kf.
22 forensic psychiatry/ or "commitment of mentally ill"/ or insanity defense/
23 (correctional or forensic).jw.
24 judicial.tw,kf.
25 (court or courts).tw,kf.
26 (prosecution? or adjudication? or sentencing or sanctioning).tw,kf.
27 ((pre-trial or pretrial or preliminary) adj hearing?).tw,kf.
28 (crime or crimes).tw,kf.
29 (justice adj2 (system? or setting?)).tw,kf.
30 or/1-29
31 guideline.pt. or exp guideline/ or exp practice guideline/ or exp Consensus/ or exp Consensus Development Conference, NIH/ or exp Consensus Development Conference/ or (consensuses or consensus or position statement or position statements or practice parameter or practice parameters or "appropriate use criteria" or appropriateness criteria or guidance statement or guidance statements or guideline or guidelines or bulletin).ti,bt.
32 30 and 31
33 32 not (exp animals/ not exp humans/)

Database: Embase Classic+Embase <1947 to 2023 March 15>
Platform: Ovid
Date searched: 2023-03-16

1 exp PRISON/
2 PRISONER/
3 Offender/
4 Criminal Justine/ or Jurisprudence/
5 legal procedure/ or probation/
6 (jurisprudenc* or ligitat*).tw,kw.
7 (legal adj (system? or servic*)).tw,kw.
8 (prisoner* or prison? or imprison*).tw,kw.
9 (inmate* or convict* or criminal* or offender?).tw,kw.
10 (correctional adj2 (setting? or service? or units or unit or facility or facilities or institution* or centre* or center*)).tw,kw.
11 (penal adj2 (setting? or service? or units or unit or facility or facilities or institution* or centre* or center*)).tw,kw.
12 (jail* or penitentiar* or gaol*).tw,kw.
13 incarcerat*.tw,kw.
14 (detain* or detention?).tw,kw.
15 parole?.tw,kw.
16 probation*.tw,kw.
17 felon*.tw,kw.
18 exp police/ or detention/
19 (police or policing).tw,kw.
20 law enforce*.tw,kw.
21 forensic*.tw,kw.
22 exp forensic medicine/
23 (correctional or forensic).jx.
24 judicial.tw,kf.
25 (court or courts).tw,kf.
26 (prosecution? or adjudication? or sentencing or sanctioning).tw,kf.
27 ((pre-trial or pretrial or preliminary) adj hearing?).tw,kf.
28 (crime or crimes).tw,kf.
29 (justice adj2 (system? or setting?)).tw,kf.
30 or/1-29
31 *practice guideline/
32 consensus/
33 consensus development/
34 (consensuses or consensus or position statement or position statements or practice parameter or practice parameters or "appropriate use criteria" or appropriateness criteria or guidance statement or guidance statements or guideline or guidelines or bulletin).ti,bt.
35 or/31-34
36 30 and 35
37 36 not medline.cr.
38 limit 37 to conference abstracts
39 37 not 38
40 39 not (((rat or rats or mouse or mice or swine or porcine or murine or sheep or lambs or pigs or piglets or rabbit or rabbits or cat or cats or dog or dogs or cattle or bovine or monkey or monkeys or trout or marmoset$1).ti. and animal experiment/) or (Animal experiment/ not (human experiment/ or human/)))

Database: APA PsycInfo <1806 to March Week 1 2023>
Platform: Ovid
Date searched: 2023-03-16

1 exp Correctional Institutions/
2 exp Prisoners/
3 Criminal Offenders/
4 exp Criminal Justice/ or Criminal Law/
5 Legal Processes/ or probation/ or Parole/
6 (jurisprudenc* or ligitat*).ti,ab.
7 (legal adj (system? or servic*)).ti,ab.
8 (prisoner* or prison? or imprison*).ti,ab.
9 (inmate* or convict* or criminal* or offender?).ti,ab.
10 (correctional adj2 (setting? or service? or units or unit or facility or facilities or institution* or centre* or center*)).ti,ab.
11 (penal adj2 (setting? or service? or units or unit or facility or facilities or institution* or centre* or center*)).ti,ab.
12 (jail* or penitentiar* or gaol*).ti,ab.
13 incarcerat*.ti,ab.
14 (detain* or detention?).ti,ab.
15 parole?.ti,ab.
16 probation*.ti,ab.
17 felon*.ti,ab.
18 Police Personnel/ or exp Law Enforcement/
19 (police or policing).ti,ab.
20 law enforce*.ti,ab.
21 forensic*.ti,ab.
22 Forensic Psychiatry/ or Forensic Psychology/
23 (correctional or forensic).jx.
24 judicial.ti,ab.
25 (court or courts).ti,ab.
26 (prosecution? or adjudication? or sentencing or sanctioning).ti,ab.
27 ((pre-trial or pretrial or preliminary) adj hearing?).ti,ab.
28 (crime or crimes).ti,ab.
29 (justice adj2 (system? or setting?)).ti,ab.
30 or/1-29
31 treatment guidelines/
32 (consensuses or consensus or position statement or position statements or practice parameter or practice parameters or "appropriate use criteria" or appropriateness criteria or guidance statement or guidance statements or guideline or guidelines or bulletin).ti,bt.
33 31 or 32
34 30 and 33
35 limit 34 to animal
36 limit 34 to human
37 34 not (35 not 36)

****

Database: CINAHL Complete
Platform: EBSCOhost
Date searched: 2023-03-16

#	Query	Limiters/Expanders
S1	(MH "Correctional Facilities")	Expanders - Apply equivalent subjects<br>Search modes - Boolean/Phrase
S2	(MH "Prisoners")	Expanders - Apply equivalent subjects<br>Search modes - Boolean/Phrase
S3	(MH "Public Offenders+")	Expanders - Apply equivalent subjects<br>Search modes - Boolean/Phrase
S4	(MH "Jurisprudence") OR (MH "Criminal Justice")	Expanders - Apply equivalent subjects<br>Search modes - Boolean/Phrase
S5	TI ((jurisprudenc* or ligitat*)) OR AB ((jurisprudenc* or ligitat*))	Expanders - Apply equivalent subjects<br>Search modes - Boolean/Phrase
S6	TI ((legal n1 (system* or servic*))) OR AB ((legal n1 (system* or servic*))) OR TI (((pre-trial or pretrial or preliminary) n1 hearing?)) OR AB (((pre-trial or pretrial or preliminary) n1 hearing?))	Expanders - Apply equivalent subjects<br>Search modes - Boolean/Phrase
S7	TI ((prison* or imprison* or jusdicial or court or courts)) OR AB ((prison* or imprison* or judicial or court or courts))	Expanders - Apply equivalent subjects<br>Search modes - Boolean/Phrase
S8	TI ((inmate* or convict* or criminal* or offender* or crime or crimes)) OR AB ((inmate* or convict* or criminal* or offender* or crime or crimes))	Expanders - Apply equivalent subjects<br>Search modes - Boolean/Phrase
S9	TI ((correctional n2 (setting or settings or service or services or units or unit or facility or facilities or institution* or centre* or center*))) OR AB ((correctional n2 (setting or settings or service or services or units or unit or facility or facilities or institution* or centre* or center*)))	Expanders - Apply equivalent subjects<br>Search modes - Boolean/Phrase
S10	TI ((penal n2 (setting or settings or service or services or units or unit or facility or facilities or institution* or centre* or center*))) OR AB ((penal n2 (setting or settings or service or services or units or unit or facility or facilities or institution* or centre* or center*)))	Expanders - Apply equivalent subjects<br>Search modes - Boolean/Phrase
S11	TI ((jail* or penitentiar* or gaol*)) OR AB ((jail* or penitentiar* or gaol*)) OR TI (justice n2 (system# or setting#)) OR AB (justice n2 (system# or setting#))	Expanders - Apply equivalent subjects<br>Search modes - Boolean/Phrase
S12	TI incarcerat* OR AB incarcerat*	Expanders - Apply equivalent subjects<br>Search modes - Boolean/Phrase
S13	TI (detain* or detention* or prosecution# or adjudication# or sentencing or sanctioning) OR AB (detain* or detention* or prosecution# or adjudication# or sentencing or sanctioning)	Expanders - Apply equivalent subjects<br>Search modes - Boolean/Phrase
S14	TI parole* OR AB parole*	Expanders - Apply equivalent subjects<br>Search modes - Boolean/Phrase
S15	TI probation* OR AB probation*	Expanders - Apply equivalent subjects<br>Search modes - Boolean/Phrase

S16	TI felon* OR AB felon*	Expanders - Apply equivalent subjects<br>Search modes - Boolean/Phrase
S17	TI (police or policing) OR AB (police or policing)	Expanders - Apply equivalent subjects<br>Search modes - Boolean/Phrase
S18	TI law enforce* OR AB law enforce*	Expanders - Apply equivalent subjects<br>Search modes - Boolean/Phrase
S19	TI forensic* OR AB forensic*	Expanders - Apply equivalent subjects<br>Search modes - Boolean/Phrase
S20	(MH "Police")	Expanders - Apply equivalent subjects<br>Search modes - Boolean/Phrase
S21	(MH "Forensic Psychiatry+")	Expanders - Apply equivalent subjects<br>Search modes - Boolean/Phrase
S22	SO correctional or forensic	Expanders - Apply equivalent subjects<br>Search modes - Boolean/Phrase
S23	TI judicial OR AB judicial	Expanders - Apply equivalent subjects<br>Search modes - Boolean/Phrase
S24	(TI court or courts) OR (AB court or courts)	Expanders - Apply equivalent subjects<br>Search modes - Boolean/Phrase
S25	TI (prosecution# or adjudication# or sentencing or sanctioning) OR AB (prosecution# or adjudication# or sentencing or sanctioning)	Expanders - Apply equivalent subjects<br>Search modes - Boolean/Phrase
S26	TI (((pre-trial or pretrial or preliminary) n1 hearing#)) OR AB (((pre-trial or pretrial or preliminary) n1 hearing#))	Expanders - Apply equivalent subjects<br>Search modes - Boolean/Phrase
S27	TI (crime or crimes) OR AB (crime or crimes)	Expanders - Apply equivalent subjects<br>Search modes - Boolean/Phrase
S28	TI ((justice n2 (system# or setting#))) OR AB ((justice n2 (system# or setting#)))	Expanders - Apply equivalent subjects<br>Search modes - Boolean/Phrase
S29	S1 OR S2 OR S3 OR S4 OR S5 OR S6 OR S7 OR S8 OR S9 OR S10 OR S11 OR S12 OR S13 OR S14 OR S15 OR S16 OR S17 OR S18 OR S19 OR S20 OR S21 OR S22 OR S23 OR S24 OR S25 OR S26 OR S27 OR S28	Expanders - Apply equivalent subjects<br>Search modes - Boolean/Phrase
S30	TI (consensuses or consensus or position statement or position statements or practice parameter or practice parameters or "appropriate use criteria" or appropriateness criteria or guidance statement or guidance statements or guideline or guidelines or bulletin)	Expanders - Apply equivalent subjects<br>Search modes - Boolean/Phrase
S31	(MH "Consensus")	Expanders - Apply equivalent subjects<br>Search modes - Boolean/Phrase
S32	(MH "Practice Guidelines")	Expanders - Apply equivalent subjects<br>Search modes - Boolean/Phrase
S33	S30 OR S31 OR S32	Expanders - Apply equivalent subjects<br>Search modes - Boolean/Phrase
S34	S29 AND S33	Expanders - Apply equivalent subjects<br>Search modes - Boolean/Phrase

Grey Literature Search Strategy

A grey literature search was conducted to identify CPGs for CJS intersection outside of the peer-reviewed literature. The followings resources were searched to identify potential CPGs.

1. Websites included in Grey Matters: A Practical Tool for Searching Health-Related Literature [1]
2. Targeted websites identified by the research team, including guideline development organizations, CPG databases/repositories, health technology assessment agencies, medical or allied health professional associations, and brain injury and housing organizations
3. First ten pages of Google Search

The reviewers documented the name and link to each website/organization and the date the website/organization was identified into an Excel file to develop a list of unique targeted websites. These websites were then searched by two independent reviewers (ZC, MJE, JMJ, or SH) for potentially relevant CPGs. The keywords and definitions listed below were used to search the websites. Where available, the search bar was used to search for grey literature; websites without a search bar were manually reviewed. The initial search for CPGs for CJS intersection was conducted in August 2022 and updated in March 2023.

Search dates, authors, titles, and links of the potentially relevant CPGs were documented in the Excel file to generate a list of unique articles for review. This list was then compared to CPGs identified through the search for peer-reviewed literature and duplicates were removed prior to screening.

The study selection process, including reasons for excluding articles, was documented in an Excel file. All articles retrieved from the targeted websites were screened by two independent reviewers from our evaluation team (ZC, MJE, JMJ, or SH) using eligibility criteria outlined in Table 1 of the manuscript.

Keywords Describing CJS Involvement or Content Consistent with the Definition of CJS Involvement

**CJS Involvement**	Keywords:<br>police or officer or arrest or offender or suspect or crime or criminal or justice trial or detention or probation or parole or legal or law or court or jail or prison or corrections or inmate or forensic<br><br>Definition of the Different Parts of CJS Involvement:<br>a) Policing (e.g., involvement with police interactions and arrest procedures)<br>b) Courts (e.g., involvement with prosecution and pretrial hearings, adjudication, and sentencing and sanctioning)<br>c) Corrections (e.g., involvement with correctional facilities, including prisons and jails)<br>d) Parole and probation (e.g., involvement with the parole or probation systems)

CJS: Criminal Justice System

The following targeted websites and databases were searched for grey literature:

- Academy of Medicine of Malaysia. Clinical Practice Guidelines [Malaysia]
- Aetna, Inc. Clinical Policy Bulletins [United States]
- Agencia de Evaluación de Tecnologías Sanitarias (AETS), Instituto de Salud Carlos III (ISCIII) [Spain]
- Agència de Qualitat i Avaluació Sanitàries de Catalunya (Agency for Health Quality and Assessment of Catalonia) [Spain]
- Agency for Healthcare Research and Quality (AHRQ) [United States]
- Alberta Health and Wellness: Decision Process Provincial Reviews [Canada]
- Alberta Medical Association. Toward Optimized Practice (TOP) [Canada]
- American Academy of Physical Medicine and Rehabilitation [United States]
- American Association for Clinical Chemistry (AACC). Practice Guidelines [United States]
- Australian Government Department of Health and Ageing. Australia and New Zealand Horizon Scanning Network (ANZHSN) [Australia]
- Australian Government Department of Health and Ageing. Medical Services Advisory Committee (MSAC) Applications. [Australia]
- Bandolier. Bandolier Knowledge [United Kingdom]
- Best Practice Advocacy Centre New Zealand (bpacNZ). bpacNZ better medicine [New Zealand]
- British Columbia Ministry of Health. BC Guidelines [Canada]
- Canadian Agency for Drugs and Technologies in Health (CADTH) [Canada]

- Canadian Alliance to End Homelessness [Canada]
- Canadian Housing First Toolkit [Canada]
- Canadian Medical Association (CMA). CMA Infobase. Clinical Practice Guidelines [Canada]
- Canadian Partnership Against Cancer. Cancer Guidelines Database [Canada]
- Canadian Standards Association (CSA). Occupational Health and Safety [Canada]
- Centers for Disease Control and Prevention (CDC). Public Health Genomics Knowledge Base. Guideline Database [United States]
- Centers for Medicare & Medicaid Services (CMS). Technology Assessments [United States]
- Centre for Urban Health Solutions [Canada]
- Centre of Evidence-Based Physiotherapy (CEBP). Physiotherapy Evidence Database: PEDro [Australia]
- Cochrane Methods Equity Homeless Health Guidelines [United Kingdom]
- Comité d'Evaluation et de Diffusion des Innovations Technologiques (CEDIT) [France]
- De Gezondheidsraad (Health Council of the Netherlands) [Netherlands]
- Deutsche Institut für Medizinische Dokumentation und Information (DIMDI) [Germany]
- Drug Safety and Effectiveness Network (DSEN) [Canada]
- ECRI Institute [United States]
- Epistemonikos [Created in Chile]
- Evidence Exchange Network for Mental Health and Addictions [Canada]
- Folkehelseinstituttet (Norwegian Institute of Public Health) Publications [Norway]
- Haute Autorité de santé (HAS) / French National Authority for Health [France]
- Health Information and Quality Authority. Health Technology Assessments [Ireland]
- Health Quality Council of Alberta (HQCA). Completed Reviews [Canada]
- Health Quality Ontario (HQO). Health Technology Assessment [Canada]
- Health Service Executive. Irish Health Repository (Lenus) [Ireland]
- Healthcare Improvement Scotland. Published Resources [Scotland]
- Institut national d’excellence en santé et en services sociaux (INESSS) [Canada]
- Institute for Clinical and Economic Review (ICER) [United States]
- Institute for Clinical Systems Improvement (ICSI). Guidelines [United States]
- Institute of Health economics (IHE). Publications [Canada]
- Institute of Technology Assessment (ITA) Projects [Austria]
- International Network of Agencies for Health Technology Assessment (INAHTA) [Canada]
- Joanna Briggs Institute (JBI) EBP Database [Australia]
- Kenniscentrum voor de Gezondheidszorg / Le Centre d'expertise des soins de santé. Belgian Health Care Knowledge Centre (KCE) [Belgium]
- Latin-American and Caribbean Center on Health Sciences Information (LILACS) [Brazil]
- Ludwig Boltzmann Institute für Health Technology Assessment (LBI) [Austria]
- Manitoba Centre for Health Policy (MCHP). Deliverables [Canada]
- McGill University Health Centre (MUHC). Technology Assessment Unit Reports [Canada]
- McMaster University, McMaster Health Forum. Health Systems Evidence [Canada]
- MD Anderson Cancer Centre [United States]
- Mental Health Commission of Canada [Canada]
- Model Systems Knowledge Translation Center [United States]

- Monash Health Centre for Clinical Effectiveness (CCE) [Australia]
- National Association of State Head Injury Administrators [United States]
- National Center for Biotechnology Information (NCBI). Bookshelf [United States]
- National Collaborating Centre for Chronic Conditions [Canada]
- National Health and Medical Research Council (NHMRC). Australia’s Clinical Practice Guidelines Portal [Australia]
- National Health Care for the Homeless Council [United States]
- National Health Service UK (NHS) [United Kingdom]
- National Institute for Health and Care Excellence (NICE) [United Kingdom]
- National Institute for Health Research (NIHR) Innovation Observatory [Australia]
- National Institute on Disability, Independent Living and Rehabilitation Research (NIDILRR) [United States]
- National Prescribing Service RADAR [Australia]
- National Rehabilitation Information Center (NARIC). REHABDATA database [United States]
- NIHR Evaluation, Trials and Studies Coordinating Centre (NETSCC) [United Kingdom]
- NLCAHR: Newfoundland and Labrador Centre for Applied Health Research. Contextualized Health Research Synthesis Program (CHRSP). Completed CHRSP projects [Canada]
- Ontario Association of Medical Laboratories (OAML) [Canada]
- Ottawa Hospital Research Institute (OHRI). Knowledge Synthesis Group [Canada]
- Programs for Assessment of Technology in Health (Canada). Reports (PATH) [Canada]
- Public Health Agency of Canada (PHAC). Disease Prevention and Control Guidelines [Canada]
- Queensland Government (Australia) Health Technology Reference Group. Health Technologies Evaluated-Reports and Briefs (COAG Health Council) [Australia]
- Registered Nurses' Association of Ontario (RNAO). Nursing Best Practice Guidelines [Canada]
- Ruff Institute of Global Homelessness [United States]
- Sahlgrenska Universitetssjukhuset (Sahlgrenska University Hospital) [Sweden]
- Scottish Intercollegiate Guidelines Network (SIGN) [Scotland]
- Sundhedsstyrelsen. Danish Health and Medicines Authority (DHMA) [Denmark]
- Swedish Council on Health Technology Assessment (SBU) [Sweden]
- Synergus. HTA Update [Sweden]
- The Alberta College of Family Physicians (ACFP). Tools for Practice [Canada]
- The Center for Brain Injury Research and Training [United States]
- The College of Physicians and Surgeons of Ontario (CPSO). CPGs & Other Guidelines [Canada]
- The Homeless Hub [Canada]
- The Hospital for Sick Children. Technology Assessment at SickKids (TASK) [Canada]
- The Regulation and Quality Improvement Authority (RQIA). Guidelines [United Kingdom]
- Therapeutics Initiative: Therapeutics Letter [Canada]
- Toronto Alliance to End Homelessness [Canada]
- Toronto Mental Health and Addictions Supporting Housing Network [Canada]

- TRIP Database (TRIP). Trip Database - Clinical Search Engine [United Kingdom]
- UK Department of Health International Resource for Infection Control (iNRIC) [United Kingdom]
- University of British Columbia Centre for Health Services and Policy Research [Canada]
- University of Ottawa. School of Rehabilitation Science. Evidence-based Practice [Canada]
- University of Queensland. OTseeker [Australia]
- University of York (CRD). Centre for Reviews and Dissemination [Canada]
- University of York. PROSPERO: International prospective register of systematic reviews [Canada]
- UpToDate. UpToDate.com [United States]
- US National Library of Medicine (NLM). PubMed. [United States]
- US National Library of Medicine & National Institutes of Health (NIH). PubMed Central [United States]
- Washington State Health Care Authority (HCA) Health Technology Review [United States]
- Wellesley Institute [Canada]
- Wiley InterScience. Cochrane Library [United Kingdom]
- Winnipeg Regional Health Authority (WRHA). Evidence Informed Practice Tools [Canada]
- World Health Organization Regional Office for Europe Health Evidence Network (WHO HEN) [Switzerland]
- Zorginstituut Nederland (National Health Care Institute Netherlands) [Netherlands]

Reference List Search Strategy

The references of all CPGs that met the eligibility criteria for this review, as well as any systematic or scoping reviews of CPGs identified during the full-text screening process, were screened to identify potential CPGs.

The initial reference list search for CPGs for CJS intersection was conducted in August 2022 and the updated reference list search was conducted in May 2023.

References

1. The Canadian Agency for Drugs and Technologies in Health (CADTH). Grey matters: A practical tool for searching health-related grey literature Ottawa: CADTH; 2018 [cited 2021 October 20]. [Available from: https://www.cadth.ca/grey-matters-practical-tool-searching-health-related-grey-literature-0]. [Accessed May 14 2024].
