# Supplementary material for: Equity considerations in clinical practice guidelines for traumatic brain injury and the criminal justice system: A systematic review
Source: PLoS Med. 2024 Aug 12;21(8):e1004418. doi: 10.1371/journal.pmed.1004418 (PMC11319042; doi:10.1371/journal.pmed.1004418)
Supplement: S1 Data — (PDF) [file pmed.1004418.s003.pdf]

S1 Data. Data Extraction and Synthesis

Table A. Text-Positive/Text-Negative Categories of Clinical Practice Guidelines for TBI

CPGs for TBI	
**Text-Positive CPGs**	Category 1: Guideline specifically recommended evidence-based diagnostic, management, or treatment approaches for individuals with CJS involvement<br><br>Category 2: Guideline acknowledged or made reference to data (e.g., epidemiologic, risk factors, outcome) regarding individuals with CJS involvement, without recommendations<br><br>Category 3: Guideline mentioned individuals with CJS involvement without context related to the literature or recommendations
**Text-Negative CPGs**	Category 1: Reference lists contained articles that included keywords for CJS involvement but the text of the guideline did not contain any keywords for or content consistent with the definition of CJS involvement<br><br>Category 2: No article in the reference list included keywords for CJS involvement

CJS: Criminal justice system; **CPGs**: Clinical practice guidelines; **TBI**: Traumatic brain injury

Table B. Data Extraction of Clinical Practice Guidelines for TBI

Guideline Name<br><br>Year<br><br>Country	Focus of the Guideline<br><br>Target Population	Text-Positive vs Text-Negative<br><br>Category	Page Reference for Data Used to Categorize Guideline (Where Applicable)	Category of CJS Intersection
**ACR Appropriateness Criteria® Head Trauma-Child [1]**<br><br>**2020**<br><br>**United States**	Diagnosis and management<br><br>Children with TBI (<16)	Text-Negative<br><br>Category 2	N/A	N/A
**ACR Appropriateness Criteria® Head Trauma: 2021 Update [2]**<br><br>**2021**<br><br>**United States**	Diagnosis and management<br><br>Adults with TBI (≥16)	Text-Negative<br><br>Category 2	N/A	N/A
**Acute Mild Traumatic Brain Injury (Concussion) in Adults [3]**<br><br>**2022**<br><br>**United States**	Evaluation and management<br><br>Adults with mTBI	Text-Negative<br><br>Category 2	N/A	N/A
**American Medical Society for Sports Medicine Position Statement on Concussion in Sport [4]**<br><br>**2018**	Diagnosis and management<br><br>Persons with sports-related concussion	Text-Negative<br><br>Category 2	N/A	N/A

**United States**				
**Assessment and Management of the Risk of Serious Neurological Complications Following Mild Traumatic Brain Injury [5]**<br><br>**2021**<br><br>**Canada**	Assessment and management of serious neurological complications<br><br>Adults and children with mTBI in the ER	Text-Negative<br><br>Category 2	N/A	N/A
**Behavioral and Affective Disorders After Brain Injury: French Guidelines for Prevention and Community [6]**<br><br>**2016**<br><br>**France**	Prevention of behavioural and affective disorders for outpatients and the identification of support systems<br><br>Adults with TBI and caregivers	Text-Positive<br><br>Category 3	Page 69	Corrections
**Beta-Blockers and Traumatic Brain Injury: A Systematic Review, Meta-Analysis, and Easter Association for the Surgery of Trauma Guideline [7]**<br><br>**2017**<br><br>**Canada and the United States**	Acute management<br><br>Adults ≥16 with acute severe TBI who are admitted to the ICU	Text-Negative<br><br>Category 2	N/A	N/A
**Care Management of the Agitation or Aggressiveness Crisis in Patients with TBI. Systematic Review of the Literature and Practice Recommendations [8]**<br><br>**2016**<br><br>**France**	Management of agitation and aggressiveness<br><br>Adults with TBI	Text-Positive<br><br>Category 3	Page 63	Corrections, Other

**Care of the Patient with Mild Traumatic Brain Injury [9]**<br><br>**2011**<br><br>**United States**	Management of mTBI<br><br>Persons with mTBI	Text-Negative<br><br>Category 2	N/A	N/A
**Centers for Disease Control and Prevention Guideline on the Diagnosis and Management of Mild Traumatic Brain Injury Among Children [10]**<br><br>**2018**<br><br>**United States**	Diagnosis, prognosis, treatment, management, and return to school<br><br>Children with mTBI (≤18)	Text-Negative<br><br>Category 2	N/A	N/A
**Clinical Policy: Critical Issues in the Management of Adult Patients Presenting to the Emergency Department with Mild Traumatic Brain Injury [11]**<br><br>**2023**<br><br>**United States**	Emergency department evaluation and management<br><br>Adults with blunt head injury or diagnosed with mild traumatic brain injury	Text-Positive<br><br>Category 2	Page e64	Corrections
**Clinical Practice Guideline for the Management of Communication and Swallowing Disorders Following Paediatric Traumatic Brain Injury [12]**<br><br>**2017**<br><br>**Australia**	Assessment, rehabilitation, and management of speech, language, and swallowing after paediatric TBI<br><br>Children (<18) with moderate or severe TBI within first year of recovery who are at risk for or are presenting with speech, language and or swallowing disorders	Text-Negative<br><br>Category 2	N/A	N/A

**Clinical Practice Guidelines for the Care of People Living with Traumatic Brain Injury in the Community [13]**<br><br>**2004**<br><br>**Australia**	Diagnosis and management<br><br>Adults with TBI from road traffic accidents	Text-Positive<br><br>Category 3	Page 51	Policing
**Clinical Practice Guidelines in Severe Traumatic Brain Injury in Taiwan [14]**<br><br>**2009**<br><br>**Taiwan**	Management<br><br>Adults and children with severe TBI	Text-Negative<br><br>Category 2	N/A	N/A
**Concussion in Children and Adolescents [15]**<br><br>**2022**<br><br>**United States**	Management<br><br>Children and adolescents with mTBI/concussion	Text-Negative<br><br>Category 2	N/A	N/A
**Concussion Management for Children Has Changed: New Pediatric Protocols Using the Latest Evidence [16]**<br><br>**2020**<br><br>**Canada**	Management, return to activity and return to school<br><br>Children and youth with concussive injury	Text-Negative<br><br>Category 2	N/A	N/A

**Early Management of Patients with a Head Injury. A National Clinical Guideline [17]**<br><br>**2009**<br><br>**Scotland**	Early management<br><br>Adults and children with TBI	Text-Negative<br><br>Category 2	N/A	N/A
**EFNS Guideline on Mild Traumatic Brain Injury: Report of an EFNS Task Force [18]**<br><br>**2012**<br><br>**Netherlands, Italy, Austria, Russia, Slovenia, Slovak Republic, Hungary & Germany**	Early management<br><br>Adults and children with mTBI	Text-Negative<br><br>Category 2	N/A	N/A
**Evidence-Based Guideline Update: Evaluation and Management of Concussion in Sports. Report of the Guideline Development Subcommittee of the American Academy of Neurology. Neurology® [19]**<br><br>**2013**<br><br>**United States**	Evaluation and management of sport concussion<br><br>Athletes with concussion/mTBI	Text-Negative<br><br>Category 2	N/A	N/A
**Guideline for Concussion/Mild Traumatic Brain Injury & Prolonged Symptoms [20]**<br><br>**2018**<br><br>**Canada**	Assessment and treatment of persistent symptoms<br><br>Adults (≥18) with mTBI/concussion	Text-Negative<br><br>Category 2	N/A	N/A

**Guideline for the Rehabilitation of Adults with Moderate to Severe TBI [21]**<br><br>**2016**<br><br>**Canada**	Assessment and management<br><br>Adults with moderate to severe TBI	Text-Positive<br><br>Category 2	Page 12	Policing, Parole
**Guidelines for Mild Traumatic Brain Injury Following a Closed Head Injury [22]**<br><br>**2008**<br><br>**Australia**	Acute and post-acute diagnosis and management<br><br>Adults (>16) at six months following mTBI (closed head injury)	Text-Positive<br><br>Category 2	Pages 7, 21, 53 – 55	Policing
**Guidelines for Prehospital Management of Traumatic Brain Injury 2nd Edition [23]**<br><br>**2008**<br><br>**United States**	Pre-hospital management<br><br>Persons with TBI (adults and children)	Text-Negative<br><br>Category 2	N/A	N/A
**Guidelines for the Management of Pediatric Severe Traumatic Brain Injury, Third Edition: Update of the Brain Trauma Foundation Guidelines [24]**<br><br>**2019**<br><br>**United States**	Management<br><br>Children with severe TBI (≤18)	Text-Negative<br><br>Category 2	N/A	N/A

**Guidelines for the Management of Severe Traumatic Brain Injury [25]**<br><br>**2016**<br><br>**United States**	Treatment interventions and evaluations<br><br>Adults with severe TBI	Text-Negative<br><br>Category 2	N/A	N/A
**Guidelines for the Pharmacologic Treatment of Neurobehavioural Sequelae of Traumatic Brain Injury [26]**<br><br>**2006**<br><br>**United States**	Pharmacologic management<br><br>Persons with TBI	Text-Positive<br><br>Category 3	Page 1474	Corrections
**Head Injury: Assessment and Early Management [27]**<br><br>**2014**<br><br>**United Kingdom**	Assessment and early management<br><br>Children, young people and adults with TBI	Text-Positive<br><br>Category 3	Page 2003	Policing
**Imaging Evidence and Recommendations for Traumatic Brain Injury: Advanced Neuro- and Neurovascular Imaging Techniques [28]**<br><br>**2015**<br><br>**United States**	Diagnosis/clinical imaging of TBI<br><br>Persons with TBI	Text-Negative<br><br>Category 2	N/A	N/A

**Imaging Evidence and Recommendations for Traumatic Brain Injury: Conventional Neuroimaging Techniques [29]**<br><br>**2015**<br><br>**United States**	Diagnosis/clinical imaging of TBI<br><br>Persons with TBI	Text-Negative<br><br>Category 2	N/A	N/A
**INCOG 2.0 Guidelines for Cognitive Rehabilitation Following Traumatic Brain Injury [30-36]**<br><br>**2023**<br><br>**Canada, Australia, & the United States**	Cognitive rehabilitation in all relevant phases of care<br><br>Adults (≥18 years) with moderate or severe TBI	Text-Positive<br><br>Category 2	Part III Page 53, Part IV Page 66	Other
**Initial Management of Closed Head Injury in Adults [37]**<br><br>**2011**<br><br>**Australia**	Early management<br><br>Adults (>16) with mild, moderate and severe TBI (closed-head injuries)	Text-Negative<br><br>Category 2	N/A	N/A
**Inter-Professional Clinical Practice Guideline for Vocational Evaluation Following Traumatic Brain Injury: A Systematic and Evidence-Based Approach [38]**<br><br>**2012**<br><br>**Canada**	Vocational evaluation<br><br>Adults with TBI (18-65 years of age)	Text-Positive<br><br>Category 1	Pages 171-172, 175	Other
**Intervention for Executive Functions After Traumatic Brain Injury: A Systematic Review,**	Management<br><br>Children and adults with TBI	Text-Negative<br><br>Category 2	N/A	N/A

**Meta-Analysis and Clinical Recommendations [39]**<br><br>**2008**<br><br>**United States**				
**Is Rest After Concussion "The Best Medicine?": Recommendations for Activity Resumption Following Concussion in Athletes, Civilians, and Military Service Members [40]**<br><br>**2013**<br><br>**Canada**	Activity resumption<br><br>Persons with mTBI	Text-Negative<br><br>Category 2	N/A	N/A
**Italian Guidelines on the Assessment and Management of Pediatric Head Injury in the Emergency Department [41]**<br><br>**2018**<br><br>**Italy**	Assessment and Management in the ED<br><br>Children (<16) with blunt head trauma	Text-Negative<br><br>Category 2	N/A	N/A
**Living Guideline for Pediatric Concussion Care [42]**<br><br>**2021**<br><br>**Canada**	Diagnosis and management<br><br>Children (5-18) with mTBI/concussion	Text-Negative<br><br>Category 2	N/A	N/A

**Management and Rehabilitation of Post-Acute Mild Traumatic Brain Injury [43]**<br><br>**2021**<br><br>**United States**	Post-acute management and rehabilitation<br><br>Adults with mTBI who are eligible for care in VA/DoD health systems (e.g., veterans, active-duty service members, national guard, reserve members, those in military academies and dependents)	Text-Negative<br><br>Category 2	N/A	N/A
**Management of Cranial Injuries Early-Phase [44]**<br><br>**2000**<br><br>**France**	Early management<br><br>Adults and children with severe TBI	Text-Negative<br><br>Category 2	N/A	N/A
**Management of Severe Traumatic Brain Injury (First 24 Hours) [45]**<br><br>**2018**<br><br>**France**	Management of severe TBI (within first 24 hours of injury)<br><br>Adults and children with severe TBI	Text-Negative<br><br>Category 2	N/A	N/A
**Mild Traumatic Brain Injury Program of Care [46]**<br><br>**2012**<br><br>**Canada**	Management of symptoms<br><br>Workers with mTBI	Text-Negative<br><br>Category 2	N/A	N/A

**Minor Head Trauma in Infants and Children: Management [47]**<br><br>**2021**<br><br>**United States**	Management<br><br>Infants and children with mTBI	Text-Negative<br><br>Category 2	N/A	N/A
**National Athletic Trainers' Association Position Statement: Management of Sport Concussion [48]**<br><br>**2014**<br><br>**United States**	Evaluation and management of sports-related concussion, including education, prevention, and return to play<br><br>Adults and children with sports-related concussion	Text-Negative<br><br>Category 2	N/A	N/A
**National Clinical Guideline for Non-Pharmacological Treatment of Long-Term Symptoms After Concussion [49]**<br><br>**2021**<br><br>**Denmark**	Management of long lasting symptoms<br><br>Adults (≥18 years) with concussions who experience long-lasting symptoms ≥4 weeks after the concussion	Text-Positive<br><br>Category 3	Pages 10-11	Policing
**Occupational Therapy Practice Guidelines for Adults with Traumatic Brain Injury [50]**<br><br>**2016**<br><br>**United States**	Management, rehabilitation, and treatment<br><br>Persons with TBI	Text-Negative<br><br>Category 2	N/A	N/A
**Physical Therapy Evaluation and Treatment After Concussion/Mild Traumatic Brain Injury [51]**<br><br>**2020**	Active rehabilitation and physical therapy evaluations	Text-Positive<br><br>Category 2	Page CPG 15	Policing

**United States**	Persons with mild TBI (≥8) years of age who have movement-related impairments from TBI			
**Post-Traumatic Headache**<br><br>**2021**<br><br>**United States**	Treatment<br><br>Persons with TBI	Text-Negative<br><br>Category 2	N/A	N/A
**Practice Guideline: Use of Quantitative EEG for the Diagnosis of Mild Traumatic Brain Injury: Report of the Guideline Committee of the American Clinical Neurophysiology Society [52]**<br><br>**2021**<br><br>**United States**	Diagnosis (using EEG)<br><br>Persons with mTBI	Text-Negative<br><br>Category 2	N/A	N/A
**Practice Parameter: Antiepileptic Drug Prophylaxis in Severe Traumatic Brain Injury [53]**<br><br>**2003**<br><br>**United States**	Pharmacological management<br><br>Adults with severe TBI	Text-Negative<br><br>Category 2	N/A	N/A
**Scandinavian Guidelines for Initial Management of Minimal, Mild and Moderate Head Injuries in Adults: An Evidence and Consensus-Based Update [54]**<br><br>**2013**<br><br>**Scandinavian Countries**	Acute management (within first 24 hours)<br><br>Adults (≥18) years with minimal, mild and moderate TBI	Text-Negative<br><br>Category 2	N/A	N/A

**Scandinavian Guidelines for Initial Management of Minor and Moderate Head Trauma in Children [55]**<br><br>**2016**<br><br>**Scandinavian Countries**	Acute management (within first 24 hours following injury)<br><br>Children <18 with mild and moderate TBI	Text-Positive<br><br>Category 2	Page 4	Other
**Severe Traumatic Brain Injury (TBI) in Children: Initial Evaluation and Management [56]**<br><br>**2021**<br><br>**United States**	Evaluation and management of sport concussion<br><br>Children with severe TBI	Text-Negative<br><br>Category 2	N/A	N/A
**Sleep-Wake Disorders in Patients with Traumatic Brain Injury [57]**<br><br>**2021**<br><br>**United States**	Evaluation and treatment of sleep-wake disorders<br><br>Persons with TBI with sleep-wake disorders	Text-Negative<br><br>Category 2	N/A	N/A
**Systematic Review and Clinical Recommendations for Healthcare Providers on the Diagnosis and Management of Mild Traumatic Brain Injury Among Children [58]**<br><br>**2016**<br><br>**United States**	Diagnosis and management<br><br>Children (<18) with mTBI	Text-Negative<br><br>Category 2	N/A	N/A
**The Screening and Management of Pituitary Dysfunction Following Traumatic Brain Injury in Adults: British Neurotrauma Group Guidance [59]**	Screening and management of pituitary dysfunction post-TBI<br><br>Adults with TBI	Text-Negative<br><br>Category 2	N/A	N/A

**2017**<br><br>**United Kingdom**				
**Traumatic Brain Injury: Diagnosis, Acute Management and Rehabilitation [60]**<br><br>**2006**<br><br>**New Zealand**	Diagnosis, screening, and post-acute rehabilitation<br><br>Children, adolescents and adults with TBI	Text-Positive<br><br>Category 2	Pages 6, 10,44, 115, 139, 145	Corrections, Other
**Updated Clinical Practice Guidelines for Concussion/Mild Traumatic Brain Injury and Persistent Symptoms [61]**<br><br>**2015**<br><br>**Canada and the United States**	Assessment and management<br><br>Adults (≥18) with mTBI and persistent symptoms	Text-Negative<br><br>Category 2	N/A	N/A
**VA/DoD Clinical Practice Guideline for the Management of Concussions-Mild Traumatic Brain Injury [62]**<br><br>**2016**<br><br>**United States**	Management at primary and specialty VA and DoD clinical settings at least 7 days after initial head injury<br><br>OEF and OIF service men and women (≥18) diagnosed with mTBI	Text-Negative<br><br>Category 2	N/A	N/A

ABIKUS: Acquired Brain Injury Knowledge Uptake Strategy; **ACR:** American College of Radiology; **CPG:** Clinical practice guideline; **CJS:** Criminal Justice System; **DBU:** Dansk Boldspil-Union (Danish Football Union); **DIF:** Danmarks Idrætsforbund (The Danish Sports Confederation); **DoD:** Department of Defense; **ED:** Emergency Department; **EEG:** Electroencephalogram; **EFNS:** European Federation of the Neurological Societies; **ER:** Emergency Room; **GP:** General Practitioner; **ICU:** Intensive Care Unit; **INCOG:** International Cognitive; **MA:** marketing authorization; **MS-TBI:** Moderate to severe traumatic brain injury; **mTBI:** mild traumatic brain injury; **NCG:** National Clinical Guideline; **NICE:** National Institute for Health and Care Excellence; **OEF:** Operation Enduring Freedom; **OIF:** Operation Iraqi Freedom; **PM&R:** Physical Medicine and Rehabilitation; **PTU:** *not specified within Danish to English translation*; **RTC:** randomized controlled trial; **ST:** Saint; **TBI:** Traumatic Brain Injury; **VA:** Veteran Affairs.

Table C. Text-Positive/Text-Negative Categories of Clinical Practice Guidelines for CJS Intersection

CPGs for CJS	
**Text-Positive CPGs**	Category 1: Guideline specifically recommended evidence-based diagnostic, management, or treatment approaches for individuals with TBI<br><br>Category 2: Guideline acknowledged or made reference to data (e.g., epidemiologic, risk factors, outcome) regarding individuals with TBI only, without recommendations<br><br>Category 3: Guideline mentioned individuals with TBI without context related to the literature or recommendations
**Text-Negative CPGs**	Category 1: Reference lists contained articles that included keywords for TBI but the text of the guideline did not contain any keywords for or content consistent with the definition of TBI<br><br>Category 2: No article in the reference list included keywords for TBI

CJS: Criminal justice system; **CPGs**: Clinical practice guidelines; **TBI**: Traumatic brain injury

Table S3D. Data Extraction of Clinical Practice Guidelines for CJS Intersection

Guideline Name<br>Year<br>Country<br>Source	Focus of the Guideline<br>Target Population	Text-Positive vs Text-Negative<br>Category	Data Used to Categorize Guideline (Where Applicable)	Category of CJS Intersection
**Consensus Statement on the Management of Hepatitis C in Australia's Prisons [63]**<br><br>**2022**<br><br>**Australia**	Diagnosis/testing, clinical management prevention, and continuity of care of prisoners living with hepatitis C<br><br>Individuals in adult and juvenile detention centres	Text-Negative<br><br>Category 2	N/A	Corrections
**Expert Recommendations for the Diagnosis and Treatment of Chronic Hepatitis C Infection in the Prison Setting [64]**<br><br>**2007**<br><br>**Spain**	Screening, diagnosis and management of chronic hepatitis C<br><br>Adults in Spanish penitentiaries	Text-Negative<br><br>Category 2	N/A	Corrections
**Mental Health of Adults in Contact with the Criminal Justice System: Identification and Management of Mental Health Problems and Integration of Care for Adults in Contact with the Criminal Justice System [65]**<br><br>**2017**<br><br>**United Kingdom**	Screening and management of mental health<br><br>Adults with mental health problems who are in contact with the criminal justice system	Text-Positive<br><br>Category 2	Page 21	Policing, Courts, Corrections, Parole

Guideline Name<br>Year<br>Country<br>Source	Focus of the Guideline<br>Target Population	Text-Positive vs Text-Negative<br>Category	Data Used to Categorize Guideline (Where Applicable)	Category of CJS Intersection
**Physical Health of People in Prison: Assessment, Diagnosis and Management of Physical Health Problems [66]**<br>**2016**<br>**United Kingdom**	Screening, diagnosis and management of physical health<br>Adults (≥18) in prisons or young offender institutions	Text-Positive<br>Category 1	Pages 21, 105, 113	Corrections
**Practice Parameter for the Assessment and Treatment of Youth in Juvenile Detention and Correctional Facilities [64]**<br>**2005**<br>**United States**	Mental health assessment and treatment<br>Youth in juvenile detention and correctional facilities	Text-Negative<br>Category 2	N/A	Corrections
**Prevention and Control of Infections with Hepatitis Viruses in Correctional Settings [67]**<br>**2003**<br>**United States**	Prevention, screening and management of hepatitis viruses<br>Inmates in juvenile and adult correctional facilities, correctional workers	Text-Negative<br>Category 2	N/A	Corrections

BP: Blood Pressure; **CJS:** Criminal Justice System; **GDG:** Guideline Development Group; **GP:** General Practitioner; **NICE:** National Institute for Health and Care Excellence; **TBI:** Traumatic Brain Injury; **STIs:** Sexually Transmitted Infections.

References

1. Ryan ME, Pruthi S, Desai NK, Falcone RA, Jr., Glenn OA, Joseph MM, et al. ACR Appropriateness Criteria® Head Trauma-Child. Journal of the American College of Radiology : JACR. 2020;17(5s):S125-s37 DOI: 10.1016/j.jacr.2020.01.026.
2. Shih RY, Burns J, Ajam AA, Broder JS, Chakraborty S, Kendi AT, et al. ACR Appropriateness Criteria® Head Trauma: 2021 Update. Journal of the American College of Radiology : JACR. 2021;18(5s):S13-s36 DOI: 10.1016/j.jacr.2021.01.006.
3. Evans R, Whitlow, CT. Acute mild traumatic brain injury (concussion) in adults. 2022.[Accessed August 18, 2022].
4. Harmon KG, Clugston JR, Dec K, Hainline B, Herring S, Kane SF, et al. American Medical Society for Sports Medicine position statement on concussion in sport. British Journal of Sports Medicine. 2019;53(4):213 DOI: 10.1136/bjsports-2018-100338.
5. National Institute of Excellence in Health and Social Services (INESSS). Assessment and management of the risk of serious neurological complications following mild traumatic brain injury. 2021.[Accessed August 18, 2022].
6. Luauté J, Hamonet J, Pradat-Diehl P. Behavioral and affective disorders after brain injury: French guidelines for prevention and community supports. Ann Phys Rehabil Med. 2016;59(1):68-73 DOI: 10.1016/j.rehab.2015.10.007.
7. Alali AS, Mukherjee K, McCredie VA, Golan E, Shah PS, Bardes JM, et al. Beta-blockers and Traumatic Brain Injury: A Systematic Review, Meta-analysis, and Eastern Association for the Surgery of Trauma Guideline. Annals of surgery. 2017;266(6):952-61 DOI: 10.1097/sla.0000000000002286.
8. Luauté J, Plantier D, Wiart L, Tell L. Care management of the agitation or aggressiveness crisis in patients with TBI. Systematic review of the literature and practice recommendations. Ann Phys Rehabil Med. 2016;59(1):58-67 DOI: 10.1016/j.rehab.2015.11.001.
9. West T, Bergman, K, Biggins, MS, French, B, Galletly, J, Hinkle, JL, & Morris, JM. Care of the patient with mild traumatic brain injury. 2011.[Accessed August 18, 2022].
10. Lumba-Brown A, Yeates KO, Sarmiento K, Breiding MJ, Haegerich TM, Gioia GA, et al. Centers for Disease Control and Prevention Guideline on the Diagnosis and Management of Mild Traumatic Brain Injury Among Children. JAMA pediatrics. 2018;172(11):e182853 DOI: 10.1001/jamapediatrics.2018.2853.
11. Physicians ACoE. Clinical Policy: Critical Issues in the Management of Adult Patients Presenting to the Emergency Department with Mild Traumatic Brain Injury. 2023.[Accessed
12. Morgan A MC, Anderson V, Waugh M-C, Cahill L, & the TBI Guideline Expert Working Committee. CLINICAL PRACTICE GUIDELINE FOR THE MANAGEMENT OF COMMUNICATION AND SWALLOWING DISORDERS FOLLOWING PAEDIATRIC TRAUMATIC BRAIN INJURY. 2017.[Accessed August 18, 2022].
13. Trevena L, Cameron, I., & Porwal, M. . Clinical Practice Guidelines for the Care of People Living with Traumatic Brain Injury in the Community. 2004.[Accessed August 18, 2022].

14. Liao KH, Chang CK, Chang HC, Chang KC, Chen CF, Chen TY, et al. Clinical practice guidelines in severe traumatic brain injury in Taiwan. Surgical neurology. 2009;72 Suppl 2:S66-73; discussion S-4 DOI: 10.1016/j.surneu.2009.07.004.
15. Meehan W, O'Brien, MJ. Concussion in children and adolescents: Management. 2022.[Accessed August 18, 2022].
16. DeMatteo C, Randall S, Falla K, Lin CY, Giglia L, Mazurek MF, et al. Concussion Management for Children Has Changed: New Pediatric Protocols Using the Latest Evidence. Clinical pediatrics. 2020;59(1):5-20 DOI: 10.1177/0009922819879457.
17. Scottish Intercollegiate Guidelines Network. Early management of patients with a head injury: A national clinical guideline. Hillside Crescent, Edinburgh; 2009.[Accessed August 18, 2022].
18. Vos PE, Battistin L, Birbamer G, Gerstenbrand F, Potapov A, Prevec T, et al. EFNS guideline on mild traumatic brain injury: report of an EFNS task force. European journal of neurology. 2002;9(3):207-19 DOI: 10.1046/j.1468-1331.2002.00407.x.
19. Giza CC, Kutcher JS, Ashwal S, Barth J, Getchius TSD, Gioia GA, et al. Evidence-based guideline update: Evaluation and management of concussion in sports. Report of the Guideline Development Subcommittee of the American Academy of Neurology. Neurology. 2013.
20. Ontario Neurotrauma Foundation. Guideline for Concussion/Mild Traumatic Brain Injury & Prolonged Symptoms. 2018.[Accessed August 18, 2022].
21. National Institute of Excellence in Health and Social Services (INESSS) ONF. CLINICAL PRACTICE GUIDELINE FOR THE REHABILITATION OF ADULTS WITH MODERATE TO SEVERE TBI. 2016.[Accessed August 18, 2022].
22. Motor Accidents Authority New South Wales. Guidelines for mild traumatic brain injury following closed head injury. 2008.[Accessed August 18, 2022].
23. Badjatia N, Carney N, Crocco TJ, Fallat ME, Hennes HM, Jagoda AS, et al. Guidelines for prehospital management of traumatic brain injury 2nd edition. Prehospital emergency care. 2008;12 Suppl 1:S1-52 DOI: 10.1080/10903120701732052.
24. Kochanek PM, Tasker RC, Carney N, Totten AM, Adelson PD, Selden NR, et al. Guidelines for the Management of Pediatric Severe Traumatic Brain Injury, Third Edition: Update of the Brain Trauma Foundation Guidelines, Executive Summary. Neurosurgery. 2019;84(6):1169-78 DOI: 10.1093/neuros/nyz051.
25. Carney N, Totten, AM, O'eilly, C, Ullman, JS, Hawryluk, GWJ, Bell, MJ, Bratton, SL, Chesnut, R, Harris, OA, Kissoon, N, Rubiano, AM, Shutter, L, Tasker, RC, Vavilala, MS, Wilberger, J, Wright, DW, Ghajar, J. . Guidelines for the Management of Severe Traumatic Brain Injury. Brain Trauma Foundation; 2016.[Accessed August 18, 2022].
26. Warden DL, Gordon B, McAllister TW, Silver JM, Barth JT, Bruns J, et al. Guidelines for the pharmacologic treatment of neurobehavioral sequelae of traumatic brain injury. Journal of neurotrauma. 2006;23(10):1468-501 DOI: 10.1089/neu.2006.23.1468.
27. National Institute for Health and Care Excellence. Head injury: assessment and early management. 2014.[Accessed August 18, 2022].

28. Wintermark M, Sanelli PC, Anzai Y, Tsiouris AJ, Whitlow CT. Imaging evidence and recommendations for traumatic brain injury: advanced neuro- and neurovascular imaging techniques. AJNR American journal of neuroradiology. 2015;36(2):E1-e11 DOI: 10.3174/ajnr.A4181.
29. Wintermark M, Sanelli PC, Anzai Y, Tsiouris AJ, Whitlow CT. Imaging evidence and recommendations for traumatic brain injury: conventional neuroimaging techniques. Journal of the American College of Radiology : JACR. 2015;12(2):e1-14 DOI: 10.1016/j.jacr.2014.10.014.
30. Bayley MT, Janzen S, Harnett A, Bragge P, Togher L, Kua A, et al. INCOG 2.0 Guidelines for Cognitive Rehabilitation Following Traumatic Brain Injury: What's Changed From 2014 to Now? The Journal of Head Trauma Rehabilitation. 2023;38(1).
31. Bayley MT, Janzen S, Harnett A, Teasell R, Patsakos E, Marshall S, et al. INCOG 2.0 Guidelines for Cognitive Rehabilitation Following Traumatic Brain Injury: Methods, Overview, and Principles. The Journal of Head Trauma Rehabilitation. 2023;38(1).
32. Jeffay E, Ponsford J, Harnett A, Janzen S, Patsakos E, Douglas J, et al. INCOG 2.0 Guidelines for Cognitive Rehabilitation Following Traumatic Brain Injury, Part III: Executive Functions. The Journal of Head Trauma Rehabilitation. 2023;38(1).
33. Ponsford J, Trevena-Peters J, Janzen S, Harnett A, Marshall S, Patsakos E, et al. INCOG 2.0 Guidelines for Cognitive Rehabilitation Following Traumatic Brain Injury, Part I: Posttraumatic Amnesia. The Journal of Head Trauma Rehabilitation. 2023;38(1).
34. Ponsford J, Velikonja D, Janzen S, Harnett A, McIntyre A, Wiseman-Hakes C, et al. INCOG 2.0 Guidelines for Cognitive Rehabilitation Following Traumatic Brain Injury, Part II: Attention and Information Processing Speed. The Journal of Head Trauma Rehabilitation. 2023;38(1).
35. Togher L, Douglas J, Turkstra LS, Welch-West P, Janzen S, Harnett A, et al. INCOG 2.0 Guidelines for Cognitive Rehabilitation Following Traumatic Brain Injury, Part IV: Cognitive-Communication and Social Cognition Disorders. The Journal of Head Trauma Rehabilitation. 2023;38(1).
36. Velikonja D, Ponsford J, Janzen S, Harnett A, Patsakos E, Kennedy M, et al. INCOG 2.0 Guidelines for Cognitive Rehabilitation Following Traumatic Brain Injury, Part V: Memory. The Journal of Head Trauma Rehabilitation. 2023;38(1).
37. New South Wales Ministry of Health. Initial management of closed head injury in adults. 2011.[Accessed August 18, 2022].
38. Stergiou-Kita M, Dawson D, Rappolt S. Inter-professional clinical practice guideline for vocational evaluation following traumatic brain injury: a systematic and evidence-based approach. Journal of occupational rehabilitation. 2012;22(2):166-81 DOI: 10.1007/s10926-011-9332-2.
39. Kennedy MR, Coelho C, Turkstra L, Ylvisaker M, Moore Sohlberg M, Yorkston K, et al. Intervention for executive functions after traumatic brain injury: a systematic review, meta-analysis and clinical recommendations. Neuropsychological rehabilitation. 2008;18(3):257-99 DOI: 10.1080/09602010701748644.

40. Silverberg ND, Iverson GL. Is rest after concussion "the best medicine?": recommendations for activity resumption following concussion in athletes, civilians, and military service members. J Head Trauma Rehabil. 2013;28(4):250-9 DOI: 10.1097/HTR.0b013e31825ad658.
41. Da Dalt L, Parri N, Amigoni A, Nocerino A, Selmin F, Manara R, et al. Italian guidelines on the assessment and management of pediatric head injury in the emergency department. Italian journal of pediatrics. 2018;44(1):7 DOI: 10.1186/s13052-017-0442-0.
42. Ontario Neurotrauma Foundation. Living Guideline for Pediatric Concussion Care. 2021.[Accessed August 18, 2022].
43. The Management and Rehabilitation of Post-Acute Mild Traumatic Brain Injury Work Group. VA/DoD CLINICAL PRACTICE GUIDELINE FOR THE MANAGEMENT AND REHABILITATION OF POST-ACUTE MILD TRAUMATIC BRAIN INJURY. 2021.[Accessed August 18, 2022].
44. French Language Neuroanesthesia and Resuscitation Association SAfF, French Society of Neurosurgery, French Society of Neuroradiology, French Language Resuscitation Society, French Society of Anesthesia and Resuscitation, French Pediatric Society, Francophone Society of Medical Emergencies,. Management of cranial injuries early-phase. 2000;81:643-8.
45. Geeraerts T, Velly L, Abdennour L, Asehnoune K, Audibert G, Bouzat P, et al. Management of severe traumatic brain injury (first 24hours). Anaesthesia, critical care & pain medicine. 2018;37(2):171-86 DOI: 10.1016/j.accpm.2017.12.001.
46. Workplace Safety and Insurance Board. Mild Traumatic Brain Injury Program of Care. 2012.[Accessed August 18, 2022].
47. Schutzman S. Minor head trauma in infants and children: Management. 2021.[Accessed August 18, 2022].
48. Broglio SP, Cantu RC, Gioia GA, Guskiewicz KM, Kutcher J, Palm M, et al. National Athletic Trainers' Association position statement: management of sport concussion. Journal of athletic training. 2014;49(2):245-65 DOI: 10.4085/1062-6050-49.1.07.
49. Rytter HM GH, Henriksen HK, Aaen N, Hartvigsen J, Hoegh M, Nisted I, Næss-Schmidt ET, Pedersen LL, Schytz HW, Thastum MM, Zerlang B, Callesen HE. National clinical guideline for non-pharmacological treatment of long-term symptoms after concussion. 2021.
50. Wheeler S. Occupational Therapy Practice Guidelines for Adults with Traumatic Brain Injury 2016 [[Available from: https://www.brainline.org/article/occupational-therapy-practice-guidelines-adults-traumatic-brain-injury]. [Accessed August 18 2022].
51. Quatman-Yates CC, Hunter-Giordano A, Shimamura KK, Landel R, Alsalaheen BA, Hanke TA, et al. Physical Therapy Evaluation and Treatment After Concussion/Mild Traumatic Brain Injury. The Journal of orthopaedic and sports physical therapy. 2020;50(4):Cpg1-cpg73 DOI: 10.2519/jospt.2020.0301.
52. Tenney JR, Gloss D, Arya R, Kaplan PW, Lesser R, Sexton V, et al. Practice Guideline: Use of Quantitative EEG for the Diagnosis of Mild Traumatic Brain Injury: Report of the Guideline Committee of the American Clinical Neurophysiology Society. Journal of clinical neurophysiology : official publication of the American Electroencephalographic Society. 2021;38(4):287-92 DOI: 10.1097/wnp.0000000000000853.

53. Chang BS, Lowenstein DH. Practice parameter: Antiepileptic drug prophylaxis in severe traumatic brain injury. Neurology. 2003;60(1):10 DOI: 10.1212/01.WNL.0000031432.05543.14.
54. Undén J, Ingebrigtsen T, Romner B, the Scandinavian Neurotrauma C. Scandinavian guidelines for initial management of minimal, mild and moderate head injuries in adults: an evidence and consensus-based update. BMC Medicine. 2013;11(1):50 DOI: 10.1186/1741-7015-11-50.
55. Astrand R, Rosenlund C, Undén J. Scandinavian guidelines for initial management of minor and moderate head trauma in children. BMC medicine. 2016;14:33 DOI: 10.1186/s12916-016-0574-x.
56. Vavilala M, Tasker, RC. . Severe traumatic brain injury (TBI) in children: Initial evaluation and management. 2021.[Accessed August 18, 2022].
57. Lim M, Baumann, CR. . Sleep-wake disorders in patients with traumatic brain injury. 2021.[Accessed August 18, 2022].
58. Pediatric mTBI Guideline Workgroup. Systematic Review and Clinical Recommendations for Healthcare Providers on the Diagnosis and Management of Mild Traumatic Brain Injury Among Children 2016.[Accessed August 18, 2022].
59. Tan CL, Alavi SA, Baldeweg SE, Belli A, Carson A, Feeney C, et al. The screening and management of pituitary dysfunction following traumatic brain injury in adults: British Neurotrauma Group guidance. Journal of neurology, neurosurgery, and psychiatry. 2017;88(11):971-81 DOI: 10.1136/jnnp-2016-315500.
60. New Zealand Guidelines Group. Traumatic brain injury: Diagnosis, acute management and rehabilitation. The Terrace, Wellington, New Zealand; 2006.[Accessed August 18, 2022].
61. Marshall S, Bayley M, McCullagh S, Velikonja D, Berrigan L, Ouchterlony D, et al. Updated clinical practice guidelines for concussion/mild traumatic brain injury and persistent symptoms. Brain Inj. 2015;29(6):688-700 DOI: 10.3109/02699052.2015.1004755.
62. The Management of Concussion-mild Traumatic Brain Injury Working Group. VA/DoD CLINICAL PRACTICE GUIDELINE FOR THE MANAGEMENT OF CONCUSSION-MILD TRAUMATIC BRAIN INJURY. Department of Veterans Affairs,n Department of Defense; 2016.[Accessed August 18, 2022].
63. Winter RJ SY, Papaluca TJ, Macdonald G, Rowland J, Colman A, Stoové M*, Lloyd AR*, Thompson AJ*, on behalf of the National Prisons Hepatitis Network,. Consensus Statement on the Management of Hepatitis C in Australia's Prisons. National Prisons Hepatitis Network (NPHN); 2022.[Accessed July 27, 2023].
64. Penn JV, Thomas C. Practice parameter for the assessment and treatment of youth in juvenile detention and correctional facilities. Journal of the American Academy of Child and Adolescent Psychiatry. 2005;44(10):1085-98 DOI: 10.1097/01.chi.0000175325.14481.21.

65. National Guideline Alliance. National Institute for Health and Care Excellence: Guidelines. Mental health of adults in contact with the criminal justice system: Identification and management of mental health problems and integration of care for adults in contact with the criminal justice system. London: National Institute for Health and Care Excellence (NICE)

Copyright © National Institute for Health and Care Excellence, 2017.; 2017.
66. National Guideline Centre. Physical Health of People in Prison: Assessment, Diagnosis and Management of Physical Health Problems. London: National Institute for Health and Care Excellence (NICE); 2016.[Accessed May 24].
67. Weinbaum C, Lyerla R, Margolis HS. Prevention and control of infections with hepatitis viruses in correctional settings. Centers for Disease Control and Prevention. MMWR Recommendations and reports : Morbidity and mortality weekly report Recommendations and reports. 2003;52(Rr-1):1-36; quiz CE1-4.
