# Supplementary material for: Equity considerations in clinical practice guidelines for traumatic brain injury and the criminal justice system: A systematic review
Source: PLoS Med. 2024 Aug 12;21(8):e1004418. doi: 10.1371/journal.pmed.1004418 (PMC11319042; doi:10.1371/journal.pmed.1004418)
Supplement: S2 Data — (PDF) [file pmed.1004418.s004.pdf]

S2 Data. Quality Appraisal Table

Table A. Quality Appraisal Table of Clinical Practice Guidelines for CJS Intersection

Evaluation Tools and Items	Practice parameter for the assessment and treatment of youth in juvenile detention and correctional facilities [1]	Expert recommendations for the diagnosis and treatment of chronic hepatitis C infection in the prison setting [2]	Prevention and control of infections with hepatitis viruses in correctional settings [3]
**Assessing Equity in Clinical Practice Guidelines [4]**			
1. Discusses the burden of disease in disadvantaged Populations	Yes	Yes	Yes
2. Discusses differences between disadvantaged and privileged populations, in terms of the biology of the disease, adherence, and baseline risks	Yes	No	Yes
3. Values [of the intervention/outcome] assessed in guideline development panels through consultations with disadvantaged populations, involvement of their caregivers, reference to relevant search, or transparent reflection	No	No	No
4. Discusses barriers to implementation in disadvantaged populations, and identifies strategies to overcome these barriers	Yes	No	Yes
5. Plans for monitoring disadvantaged groups according to PROGRESS-plus elements	Yes	No	Yes
**GRADE Equity Guideline: Equity Extension of the Guideline Development [5-8]**			

Evaluation Tools and Items	Practice parameter for the assessment and treatment of youth in juvenile detention and correctional facilities [1]	Expert recommendations for the diagnosis and treatment of chronic hepatitis C infection in the prison setting [2]	Prevention and control of infections with hepatitis viruses in correctional settings [3]
**1. Setting priorities**			
a. Dedicates part of or entire guideline to the care of disadvantaged populations	Yes	Yes	Yes
**2. Guideline group membership**			
a. Includes representatives of the disadvantaged populations in the different guideline groups, particularly the voting panel	No	No	No
b. Ensures the method for recruitment of group members considers representatives of all relevant disadvantaged populations	Not reported	Not reported	Not reported
c. Recruits a methodologist who is familiar with and mindful of equity issues	Not reported	Not reported	Not reported
d. Ensures the chair of the voting panel is familiar with equity issues	Not reported	Not reported	Not reported
**3. Identifying the target audience(s)**			
a. Specifies relevant disadvantaged populations when identifying the target audience(s)	No	No	No
b. Involves representatives of the disadvantaged populations when identifying the target audience(s)	Not reported	Not reported	Not reported
**4. Generating the guideline questions**			
a. Considers equity when specifying elements of the PICO questions	Not reported	Not reported	Not reported

Evaluation Tools and Items	Practice parameter for the assessment and treatment of youth in juvenile detention and correctional facilities [1]	Expert recommendations for the diagnosis and treatment of chronic hepatitis C infection in the prison setting [2]	Prevention and control of infections with hepatitis viruses in correctional settings [3]
b. Considers "good-practice statements" that could help address equity issues	No	No	No
**5. Considers the importance of outcomes and interventions**			
a. Involves representatives of disadvantaged populations in rating the importance of interventions and outcomes	No	No	No
b. Searches selected databases for outcomes rated as important by disadvantaged populations	No	No	Not reported
c. Considers separate recommendations for disadvantaged populations if their values and preferences are thought to differ substantively to the point of affecting the strength and/or direction of the recommendation	Yes	Yes	Yes
**6. Deciding what evidence to include and searching for evidence**			
a. Seeks evidence specific to disadvantaged populations, for example, baseline risks specific to those groups	Yes	Yes	Yes
b. Considers including evidence derived from fields other than health (e.g., social science) that address disadvantaged populations	No	No	Not reported
c. Searches literature published in the language relevant to the disadvantaged population	Not reported	Not reported	Not reported

**Evaluation Tools and Items**	Practice parameter for the assessment and treatment of youth in juvenile detention and correctional facilities [1]	Expert recommendations for the diagnosis and treatment of chronic hepatitis C infection in the prison setting [2]	Prevention and control of infections with hepatitis viruses in correctional settings [3]
**7. Summarizing the evidence and considering additional information**			
a. Considers the PROGRESS-plus elements when synthesizing the evidence	Yes	Yes	Yes
b. Follows the PRISMA-equity statement when reporting the systematic reviews	Not reported	Not reported	Not reported
c. Considers information on resource use, cost, effect on equity, feasibility, and acceptability from the perspective of disadvantaged populations	Yes	Yes	Yes
**8. Wording of recommendations**			
a. Specific in defining the population to maximize the understanding that it applies to a disadvantaged population (when applicable)	Yes	Yes	Yes
b. Includes the necessary remarks following the recommendation to ensure its appropriate implementation in disadvantaged populations	Yes	Yes	Yes
c. Ensures that the language is used carefully so that the recommendation does Not stigmatize already disadvantaged populations	Yes	Yes	Yes
**9. Evaluation and use**			
a. Produces tools to facilitate implementation and use among disadvantaged populations	No	No	No

Evaluation Tools and Items	Practice parameter for the assessment and treatment of youth in juvenile detention and correctional facilities [1]	Expert recommendations for the diagnosis and treatment of chronic hepatitis C infection in the prison setting [2]	Prevention and control of infections with hepatitis viruses in correctional settings [3]
b. Monitors and audits implementation and use among disadvantaged populations	No	No	No

Evaluation Tools and Items	Physical Health of People in Prison: Assessment, Diagnosis and Management of Physical Health Problems [9]	Mental health of adults in contact with the criminal justice system: Identification and management of mental health problems and integration of care for adults in contact with the criminal justice system [10]	Consensus Statement on the Management of Hepatitis C in Australia's Prisons [11]
**Assessing Equity in Clinical Practice Guidelines [4]**			
1. Discusses the burden of disease in disadvantaged Populations	No	Yes	Yes
2. Discusses differences between disadvantaged and privileged populations, in terms of the biology of the disease, adherence, and baseline risks	No	Yes	Yes
3. Values [of the intervention/outcome] assessed in guideline development panels through consultations with disadvantaged populations, involvement of their caregivers,	Yes	Yes	No

Evaluation Tools and Items	Physical Health of People in Prison: Assessment, Diagnosis and Management of Physical Health Problems [9]	Mental health of adults in contact with the criminal justice system: Identification and management of mental health problems and integration of care for adults in contact with the criminal justice system [10]	Consensus Statement on the Management of Hepatitis C in Australia's Prisons [11]
reference to relevant search, or transparent reflection			
4. Discusses barriers to implementation in disadvantaged populations, and identifies strategies to overcome these barriers	Yes	Yes	Yes
5. Plans for monitoring disadvantaged groups according to PROGRESS-plus elements	Yes	Yes	Yes
**GRADE Equity Guideline: Equity Extension of the Guideline Development [5-8]**			
**1. Setting priorities**			
a. Dedicates part of or entire guideline to the care of disadvantaged populations	Yes	Yes	Yes
**2. Guideline group membership**			
a. Includes representatives of the disadvantaged populations in the different guideline groups, particularly the voting panel	Yes	Yes	No
b. Ensures the method for recruitment of group members considers representatives of all relevant disadvantaged populations	Not reported	No	No
c. Recruits a methodologist who is familiar with and mindful of equity issues	Not reported	Not reported	Not reported
d. Ensures the chair of the voting panel is familiar with equity issues	Not reported	Not reported	Not reported

Evaluation Tools and Items	Physical Health of People in Prison: Assessment, Diagnosis and Management of Physical Health Problems [9]	Mental health of adults in contact with the criminal justice system: Identification and management of mental health problems and integration of care for adults in contact with the criminal justice system [10]	Consensus Statement on the Management of Hepatitis C in Australia's Prisons [11]
**3. Identifying the target audience(s)**			
a. Specifies relevant disadvantaged populations when identifying the target audience(s)	Yes	Yes	No
b. Involves representatives of the disadvantaged populations when identifying the target audience(s)	Not reported	Not reported	Not reported
**4. Generating the guideline questions**			
a. Considers equity when specifying elements of the PICO questions	Yes	Yes	Not reported
b. Considers "good-practice statements" that could help address equity issues	No	No	Yes
**5. Considers the importance of outcomes and interventions**			
a. Involves representatives of disadvantaged populations in rating the importance of interventions and outcomes	Yes	Yes	No
b. Searches selected databases for outcomes rated as important by disadvantaged populations	Yes	Yes	Not reported
c. Considers separate recommendations for disadvantaged populations if their values and preferences are thought to differ substantively to the point of affecting	Yes	Yes	Yes

Evaluation Tools and Items	Physical Health of People in Prison: Assessment, Diagnosis and Management of Physical Health Problems [9]	Mental health of adults in contact with the criminal justice system: Identification and management of mental health problems and integration of care for adults in contact with the criminal justice system [10]	Consensus Statement on the Management of Hepatitis C in Australia's Prisons [11]
the strength and/or direction of the recommendation			
**6. Deciding what evidence to include and searching for evidence**			
a. Seeks evidence specific to disadvantaged populations, for example, baseline risks specific to those groups	Yes	Yes	Not reported
b. Considers including evidence derived from fields other than health (e.g., social science) that address disadvantaged populations	Yes	Yes	Not reported
c. Searches literature published in the language relevant to the disadvantaged population	No	No	Not reported
**7. Summarizing the evidence and considering additional information**			
a. Considers the PROGRESS-plus elements when synthesizing the evidence	Yes	Yes	Yes
b. Follows the PRISMA-equity statement when reporting the systematic reviews	Not reported	Not reported	Not reported
c. Considers information on resource use, cost, effect on equity, feasibility, and acceptability from the perspective of disadvantaged populations	Yes	Yes	No
**8. Wording of recommendations**			

Evaluation Tools and Items	Physical Health of People in Prison: Assessment, Diagnosis and Management of Physical Health Problems [9]	Mental health of adults in contact with the criminal justice system: Identification and management of mental health problems and integration of care for adults in contact with the criminal justice system [10]	Consensus Statement on the Management of Hepatitis C in Australia's Prisons [11]
a. Specific in defining the population to maximize the understanding that it applies to a disadvantaged population (when applicable)	Yes	Yes	Yes
b. Includes the necessary remarks following the recommendation to ensure its appropriate implementation in disadvantaged populations	Yes	Yes	No
c. Ensures that the language is used carefully so that the recommendation does Not stigmatize already disadvantaged populations	Yes	Yes	Yes
**9. Evaluation and use**			
a. Produces tools to facilitate implementation and use among disadvantaged populations	Yes	Yes	No
b. Monitors and audits implementation and use among disadvantaged populations	No	Yes	No

Response Options: N/A (Not Applicable), No, Not Reported, Yes

CJS: Criminal Justice System; **GRADE:** Grading of Recommendations Assessment, Development and Evaluation; **PICO:** Patient/population, intervention, comparison and outcomes; **PRISMA:** Preferred Reporting Items for Systematic Reviews and Meta-Analyses; **PROGRESS:** Place of residence, Race/ethnicity/culture/language, Occupation, Gender/sex, Religion, Education, Socioeconomic status, Social capital.

References

1. Penn JV, Thomas C. Practice parameter for the assessment and treatment of youth in juvenile detention and correctional facilities. Journal of the American Academy of Child and Adolescent Psychiatry. 2005;44(10):1085-98 DOI: 10.1097/01.chi.0000175325.14481.21.
2. Saiz de la Hoya-Zamácola P, Marco-Mouriño A, Clemente-Ricote G, Portilla-Sogorb J, Boix-Martínez V, Núñez-Martínez O, et al. [Expert recommendations for the diagnosis and treatment of chronic hepatitis C infection in the prison setting]. Enfermedades infecciosas y microbiologia clinica. 2006;24(9):568-75 DOI: 10.1157/13093878.
3. Weinbaum C, Lyerla R, Margolis HS. Prevention and control of infections with hepatitis viruses in correctional settings. Centers for Disease Control and Prevention. MMWR Recommendations and reports : Morbidity and mortality weekly report Recommendations and reports. 2003;52(Rr-1):1-36; quiz CE1-4.
4. Dans AM, Dans L, Oxman AD, Robinson V, Acuin J, Tugwell P, et al. Assessing equity in clinical practice guidelines. Journal of clinical epidemiology. 2007;60(6):540-6 DOI: 10.1016/j.jclinepi.2006.10.008.
5. Akl EA, Welch V, Pottie K, Eslava-Schmalbach J, Darzi A, Sola I, et al. GRADE equity guidelines 2: considering health equity in GRADE guideline development: equity extension of the guideline development checklist. Journal of clinical epidemiology. 2017;90:68-75 DOI: 10.1016/j.jclinepi.2017.01.017.
6. Pottie K, Welch V, Morton R, Akl EA, Eslava-Schmalbach JH, Katikireddi V, et al. GRADE equity guidelines 4: considering health equity in GRADE guideline development: evidence to decision process. Journal of clinical epidemiology. 2017;90:84-91 DOI: https://doi.org/10.1016/j.jclinepi.2017.08.001.
7. Welch VA, Akl EA, Guyatt G, Pottie K, Eslava-Schmalbach J, Ansari MT, et al. GRADE equity guidelines 1: considering health equity in GRADE guideline development: introduction and rationale. Journal of clinical epidemiology. 2017;90:59-67 DOI: 10.1016/j.jclinepi.2017.01.014.
8. Welch VA, Akl EA, Pottie K, Ansari MT, Briel M, Christensen R, et al. GRADE equity guidelines 3: considering health equity in GRADE guideline development: rating the certainty of synthesized evidence. Journal of clinical epidemiology. 2017;90:76-83 DOI: https://doi.org/10.1016/j.jclinepi.2017.01.015.
9. National Guideline Centre. Physical Health of People in Prison: Assessment, Diagnosis and Management of Physical Health Problems. London: National Institute for Health and Care Excellence (NICE); 2016.[Accessed May 24].
10. National Guideline Alliance. National Institute for Health and Care Excellence: Guidelines. Mental health of adults in contact with the criminal justice system: Identification and management of mental health problems and integration of care for adults in contact with the criminal justice system. London: National Institute for Health and Care Excellence (NICE)

Copyright © National Institute for Health and Care Excellence, 2017.; 2017.

11. Winter RJ SY, Papaluca TJ, Macdonald G, Rowland J, Colman A, Stoové M*, Lloyd AR*, Thompson AJ*, on behalf of the National Prisons Hepatitis Network,. Consensus Statement on the Management of Hepatitis C in Australia's Prisons. National Prisons Hepatitis Network (NPHN); 2022.[Accessed July 27, 2023].
